# Evaluating the cultural alignment of multilingual LLMs in typical Japanese workplace scenarios

**DOI:** 10.1371/journal.pone.0338524

**Published:** 2026-07-27

**Authors:** Zhiwei Gao, Nobuyuki Shimizu, Sumio Fujita, Shaowen Peng, Shoko Wakamiya, Eiji Aramaki

**Affiliations:** 1 Nara Institute of Science and Technology, Ikoma, Japan; 2 LY Corporation, Tokyo, Japan; A’Sharqiyah University, OMAN

## Abstract

While current evaluations of LLM cultural alignment predominantly rely on static benchmarks in Western contexts, their ability to navigate generative, high-context socio-pragmatic demands in non-Western environments remains critically underexplored. This study investigates how multilingual LLMs adapt to the Japanese workplace—a stringent stress-test environment characterized by strong high-context communication norms and rigid honorific conventions—using Hofstede’s six cultural dimensions as a heuristic framework. We evaluated five state-of-the-art LLMs (LLM-jp, Phi, Llama, Qwen, and GLM) through a large-scale crowdsourced human evaluation. Based on 1,718 valid evaluator sessions, native Japanese raters assessed model outputs to generate a holistic Japanese Workplace Cultural Alignment Score (JWCAS). To dissect the underlying communicative strategies, we paired this with a three-layer diagnostic sub-score analysis (Linguistic Form, Socio-Cultural Values, and Social Action). Our results reveal that leading multilingual models (Phi and GLM) achieved overall JWCAS scores comparable to, or significantly higher than, the native Japanese model (LLM-jp). Crucially, our sub-score analysis demonstrates that holistic evaluation metrics can obscure deep pragmatic deficits: while LLM-jp overfits to surface-level linguistic politeness (Layer 1), it shows critical weaknesses in socio-cultural values (Layer 2) and context-aware social strategies (Layer 3). In contrast, leading multilingual models demonstrate balanced competence across all layers. These findings suggest that true cultural competence requires moving beyond native linguistic mastery, highlighting the necessity of multi-dimensional diagnostic frameworks for cross-cultural AI alignment.

## Introduction

Large Language Models (LLMs) have revolutionized natural language processing (NLP), achieving remarkable performance in tasks such as generation, summarization, and translation. However, as these models are increasingly deployed in multilingual and cross-cultural contexts, there are concerns about whether they can correctly reflect local ways of communicating. While LLMs demonstrate strong linguistic capabilities, this does not guarantee that their outputs are socially or culturally appropriate. Prior studies suggest that LLMs often reflect the dominant cultural patterns present in their training data—frequently favoring Western-centric values in multilingual settings [1,2]. This misalignment may lead to outputs that violate implicit norms in non-Western cultures, particularly in high-context environments such as the Japanese workplace. In professional settings, this misalignment can perpetuate interpersonal friction and professional inequities.

In these contexts, interpersonal communication depends not only on what is said, but also on how it is said—taking into account hierarchy, indirectness, and emotional restraint [3].

To investigate cultural alignment, Hofstede’s Cultural Dimensions Theory offers a robust theoretical foundation for modeling national value systems. It outlines six key dimensions—Power Distance (PDI), Individualism vs. Collectivism (IDV), Uncertainty Avoidance (UAI), Masculinity vs. Femininity (MAS), Long-Term Orientation (LTO), and Indulgence vs. Restraint (IND)—that capture the socioemotional underpinnings of workplace and interpersonal behavior [4]. We acknowledge that this model has faced scholarly critique for potential cultural essentialism and for treating national cultures as static monoliths [5]. However, within the context of computational evaluation, we utilize it not as an absolute psychological truth, but as a widely adopted, quantifiable heuristic framework that provides a structured taxonomy for cross-cultural comparisons. While this framework has been widely used in psychology, management, and communication studies, its application to LLMs remains limited. Table 1 presents a brief description of each dimension in Hofstede’s cultural dimension theory.

**Table 1:** Descriptions for Hofstede’s Cultural Dimensions Theory, which provided the theoretical reference for this study.

Dimension	Description
Power Distance (**PDI**)	The extent to which less powerful members of a society accept and expect unequal distribution of power.
Individualism vs. Collectivism (**IDV**)	The degree to which individuals are integrated into groups and value personal independence over group cohesion.
Uncertainty Avoidance (**UAI**)	The extent to which people feel uncomfortable with uncertainty and ambiguity, leading to reliance on rules and stability.
Masculinity vs. Femininity (**MAS**)	The degree to which a culture values competitiveness, achievement, and material success over care, cooperation, and quality of life.
Long-Term vs. Short-Term Orientation (**LTO**)	The extent to which a society maintains long-term traditions and future-oriented values rather than focusing on short-term results.
Indulgence vs. Restraint (**IND**)	The degree to which a culture allows relatively free gratification of human desires related to enjoying life and having fun.

To ground our analysis in a clearly defined social context, we focus on the Japanese workplace rather than general Japanese culture. The workplace is a highly structured domain where cultural values are prominently and consistently expressed [6]. More importantly, the Japanese workplace serves as a uniquely rigorous stress-test environment for cross-cultural LLM evaluation. While Japan is widely recognized as a quintessential high-context culture demanding heavy reliance on implicit consensus and unstated social cues (i.e., *Kuuki wo yomu* or “reading the air”) [3,7], it simultaneously operates on a highly grammaticalized honorific system (*Keigo*) that strictly encodes social hierarchies and relational distance [8,9]. This unique dichotomy between rigid linguistic form and implicit socio-pragmatic intent provides an unparalleled lens for our study. It allows us to explicitly disentangle an LLM’s surface-level linguistic fluency from its true cultural competence, effectively exposing instances where models generate grammatically flawless text but still produce socio-pragmatically inappropriate responses [10].

Existing research on LLM cultural bias has highlighted disparities in how models reflect societal values. For example, Yanaka et al. [11] identified social biases in Japanese LLMs, linking them to training data embedded with societal stereotypes, while Naous et al. [12] measured cultural bias in multilingual models, revealing a preference for Western norms in Arabic contexts. Studies applying Hofstede’s dimensions, such as Masoud et al. [13] and Kharchenko et al. [14], have shown that while LLMs can mimic cultural values, they often reinforce training data stereotypes. These findings underscore the need for systematic evaluation of how LLMs encode cultural norms across dimensions, especially in professional settings where misalignment could perpetuate inequities.

Taken together, this prior work highlights the need for systematic evaluation, particularly concerning the gap between an LLM’s linguistic fluency and its cultural competence. We conceptualize this as the distinction between two approaches:

- **A one-size-fits-all approach**: The rigid application of a single, learned cultural style regardless of the situation.
- **A context-aware strategy**: The ability to flexibly adjust communicative strategies to meet situational demands.

For instance, a “one-size-fits-all” model might default to using profound apologies in every situation due to a superficial understanding of Japanese politeness, whereas a “context-aware” model would adjust the degree of apology or use indirect phrasing based on whether the interlocutor is a close colleague or a senior executive.

To investigate this distinction, this study evaluates the cultural alignment of five state-of-the-art multilingual LLMs: LLM-jp [15] (developed in Japan), Phi [16] and Llama [17] (primarily developed in English-speaking contexts), and Qwen [18] and GLM [19] (primarily developed in Chinese contexts) in simulated Japanese workplace scenarios. We employ a quantitative evaluation incorporating a diagnostic sub-score analysis: (1) Japanese human raters provide scores yielding the overall Japanese Workplace Cultural Alignment Score (JWCAS), while (2) a diagnostic analysis of sub-scores, grounded in communicative competence theory [20–22], dissects the specific strategies driving these scores. This integration allows us to measure the degree of alignment while simultaneously analyzing the *nature* of the strategy used (e.g., mastery of linguistic form vs. socio-cultural values vs. strategic action), potentially revealing aspects of cultural competence mechanisms.

**Fig 1:**
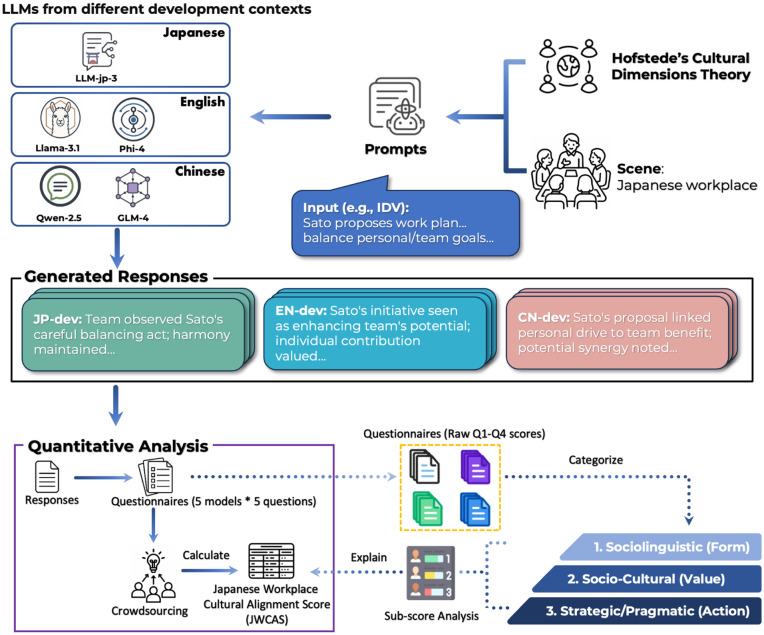
Overall workflow of the study. Japanese responses are generated from five multilingual LLMs using open-ended, culture-sensitive prompts. The evaluation employs two levels of quantitative analysis: (1) a calculation of the overall Japanese Workplace Cultural Alignment Score (JWCAS) based on holistic human ratings, and (2) a diagnostic sub-score analysis of specific questionnaire items grounded in communicative competence theory, to explain the JWCAS results.

Crucially, our findings demonstrate that holistic evaluation metrics can obscure deep pragmatic deficits. While leading multilingual models (Phi and GLM) achieved overall cultural alignment scores that rivaled or even significantly surpassed the native model (LLM-jp), our layer-by-layer diagnosis revealed that the native model heavily relies on superficial linguistic politeness (overfitting to forms), whereas multilingual models exhibit robust socio-pragmatic execution. This study underscores the necessity of moving beyond single-metric evaluations to uncover the true mechanisms driving cross-cultural alignment. Our overall workflow is illustrated in Fig 1.

## Methods

To evaluate the cultural competence of LLMs in Japanese workplace settings, our framework incorporates two levels of quantitative analysis based on human evaluations. The primary component involves calculating the overall Japanese Workplace Cultural Alignment Score (JWCAS) from holistic ratings (Q5) provided by Japanese participants. This is complemented by a diagnostic sub-score analysis, which examines ratings on specific questionnaire items (Q1-Q4) through the lens of communicative competence theory [20–22] to provide a mechanistic explanation for the JWCAS results.

### Model selection and prompting strategy

In this study, we selected five state-of-the-art multilingual LLMs developed in different regions to ensure a comprehensive analysis of cultural alignment. Model origin does not necessarily determine cultural alignment, which is likely primarily influenced by training data and fine-tuning methodologies. These models were chosen based on their diverse training backgrounds and publicly available documentation, reflecting a wide range of linguistic and cultural features. Specifically, we included **LLM-jp-3-13B**, developed in Japan and known for its proficiency in Japanese [15]. From models primarily developed in English-speaking contexts, we selected **Phi-4-14B** [16] and **Llama-3.1-8B** [17], known for generating contextually relevant responses [23]. In addition, we incorporated **Qwen-2.5-14B** [18] and **GLM-4-9B** [19], representing models primarily developed in Chinese contexts.

To ensure robust evaluation across diverse contexts, we constructed a total of 30 distinct evaluation scenarios (5 unique scenarios for each of the 6 Hofstede dimensions). To illustrate the evaluation task, consider a scenario designed to assess Power Distance: the model is prompted to role-play a junior employee who notices a significant data error in a senior colleague’s report right as a project review meeting is ending. A culturally aligned, “context-aware” response in the Japanese workplace requires wrapping the correction in indirect phrasing or choosing to address it privately after the meeting to preserve the senior colleague’s face (maintaining harmony). Conversely, a response exhibiting cultural misalignment might bluntly point out the error in front of others; even if it employs flawless polite grammar (*Keigo*), it fails the underlying socio-pragmatic requirement. The complete list of these 30 input scenarios, along with their English translations, is provided in S1 Table.

All responses generated from these 30 scenarios were subsequently evaluated using a unified set of 7 diagnostic categories, which are detailed later in Table 2.

**Table 2:** Three-Layer Communicative Competence Framework and Questionnaire Item Categorization.

Competence Layer	Diagnostic Category	Dimensions	Description (Measured Function)
**1. Sociolinguistic**	Linguistic Form	PDI(Q1, Q3)	Measures grasp of correct language, politeness forms, and hierarchical nuances (e.g., *Keigo*).
**2. Socio-Cultural**	Value Alignment	IDV(Q3, Q4), UAI(Q1, Q4), MAS(Q1, Q3)	Measures alignment with underlying cultural norms and attitudes (e.g., collectivism, uncertainty avoidance, gender roles).
	Value & Process	LTO(Q2, Q3)	Measures alignment with cultural values related to process (e.g., long-term orientation, cautious analysis).
	Value & Action	IND(Q2, Q3)	Measures alignment with cultural values related to action (e.g., restraint, modesty).
**3. Strategic/Pragmatic**	Social Strategy	PDI(Q2, Q4)	Measures execution of a specific social action (e.g., humbly accepting advice, promoting communication).
	Strategic Stance	IDV(Q1, Q2), MAS(Q2, Q4), LTO(Q1, Q4), IND(Q1, Q4)	Measures the ability to state a balanced, culturally-appropriate strategic position (e.g., balancing team vs. individual).
	Strategic Action	UAI(Q2, Q3)	Measures the ability to propose a concrete, culturally-appropriate action (e.g., proposing clear steps vs. flexible adaptation).

**Table notes:** This table maps each diagnostic category (derived from questionnaire items Q1–Q4) to its corresponding theoretical layer of communicative competence. This framework provides the basis for the sub-score analysis in the Results and Discussion sections.

This multi-scenario approach aimed to capture a wider range of relevant cultural behaviors. Prompt development involved iterative drafting, review by native Japanese speakers familiar with corporate environments to ensure authenticity, and pilot testing for clarity.

The prompts were designed to be open-ended, avoiding fixed formats to encourage natural responses reflecting implicit cultural understanding, social appropriateness, and interpersonal behavior. All prompts were written in Japanese for linguistic consistency and authenticity. Using these five prompts per dimension, each of the five models generated responses, resulting in a total corpus of 1,000 responses per model per dimension. This ensured coverage of diverse scenarios within each cultural aspect.

To conduct the crowdsourced human evaluation, responses were dynamically and randomly sampled from this larger pool of 30,000 generated texts for each evaluator. This stratified random sampling approach yielded a final set of 1,718 valid human-evaluated participant sessions. Because a single participant session involved sequentially evaluating the outputs of all five models (each presented independently one at a time) across the five questionnaire items (Q1-Q5), this resulted in a robust final dataset of 42,950 individual Likert-scale rating data points. The detailed distribution of these evaluation sessions and data points across the six Hofstede dimensions is provided in S4 Table.

To ensure reproducibility, a consistent set of hyperparameters was used for generating responses: temperature of 0.3, top-p sampling of 0.8, repetition penalty of 1.1, and a fixed random seed of 42.

### Quantitative evaluation with questionnaire

This subsection outlines our quantitative evaluation method, which is based on a structured questionnaire.

#### Questionnaire design.

The questionnaires were developed based on Hofstede’s six cultural dimensions to capture culturally salient features in Japanese workplace communication. To ensure content validity, questionnaire items were created to directly operationalize the definitions of each dimension [24], following established methodological guidelines for measure development in organizational research [25]. Each questionnaire contains five Likert-scale items (1: Strongly Disagree to 5: Strongly Agree), assessing key cultural aspects reflected in LLM responses. The internal consistency for the items in each dimension was high, as indicated by *Cronbach’s Alpha* scores: PDI (α=.95), IDV (α=.94), UAI (α=.95), MAS (α=.91), LTO (α=.94), and IND (α=.95). A pilot study was conducted with a small group of participants to refine item wording for clarity and comprehensibility before the main survey was deployed.

All questionnaire items are provided in S2 Table in the Supporting Information for reader convenience.

#### Evaluation procedure.

We recruited participants through the Yahoo! Japan Crowdsourcing platform. To ensure the cultural validity of our evaluations, participants were pre-screened based on their familiarity with Japanese workplace culture, with a requirement of having at least one year of full-time work experience in a Japanese company. During the evaluation task, each trial presented participants with a specific workplace scenario prompt and one anonymized LLM-generated response. The model identity was hidden to prevent brand bias. Within each session, participants sequentially completed multiple independent trials, in which the outputs of all five models were evaluated one at a time using the five questionnaire items (Q1–Q5). We collected data from a total of 1,718 valid participant sessions, yielding a final dataset of 42,950 individual Likert-scale rating data points for statistical modeling.

The age distribution of participants was as follows: 0.2% were aged 18–20, 4.4% were aged 21–30, 15.3% were aged 31–40, 29.5% were aged 41–50, 34.3% were aged 51–60, and 16.2% were aged 61 or older. The gender distribution was 57.9% male and 42.0% female.

#### Calculation of Japanese workplace cultural alignment score (JWCAS).

After collecting all questionnaire results, we propose the Japanese Workplace Cultural Alignment Score (JWCAS) to quantify the overall performance of each model. This score is calculated as the equally-weighted average of the mean holistic scores (${\bar{S}}_{i}^{m}$) from each of the six Hofstede dimensions.

Specifically, for a given model *m*, we first compute the average human score ${\bar{S}}_{i}^{m}$ for each dimension i∈{PDI,IDV,UAI,MAS,LTO,IND}. This score is based on the responses to questionnaire item Q5 (holistic cultural fit rating; for details, please refer to S2 Table) for that dimension.

The final JWCAS score is then calculated as the simple arithmetic mean:


$$\begin{array}{c} {\text{JWCAS}}_{m}=\frac{1}{6}\sum_{i=1}^{6}{\bar{S}}_{i}^{m} \end{array}$$ (1)

where ${\bar{S}}_{i}^{m}$ represents the average score given to the model *m* on questionnaire item Q5 for dimension *i* by participants.

#### Statistical analysis.

The human evaluation scores were analyzed using a Linear Mixed-Effects Model (LMM), selected to appropriately handle the repeated-measures design of our study. Although 5-point Likert ratings are inherently ordinal, we treated them as approximately continuous variables in the LMM as a pragmatic modeling approximation. This approach allowed us to model the repeated-measures structure of the data while accounting for evaluator- and scenario-level variation. Specifically, the LLM source (e.g., GLM, LLM-jp) was set as the fixed effect, while evaluator ID and scenario ID were included as random intercepts to account for individual rater biases and scenario-specific variation. The overall significance of the LLM source effect was first assessed using an F-test.

After the overall LMM effect was confirmed, post-hoc pairwise model comparisons were conducted using Wald-type contrasts derived from the fixed-effect estimates and their covariance matrix. Two-tailed p-values were computed from the resulting z-statistics, and the Benjamini–Hochberg false discovery rate (FDR) procedure was applied to correct for multiple pairwise comparisons [26].

Furthermore, we calculated the Intraclass Correlation Coefficient (ICC) from the LMM’s variance components to measure inter-rater reliability. The resulting ICC was 0.837, indicating *good reliability* in the evaluations [27].

### Diagnostic sub-score analysis

To provide a mechanistic explanation for the overall JWCAS performance, we conducted a direct analysis of our questionnaire’s diagnostic sub-scores. Our categorization of these items is grounded in foundational models of **Communicative Competence** [20–22], which posit that cultural adaptation requires more than formal linguistic knowledge.

We argue that overall cultural fit (measured by questionnaire item Q5) is a function of a model’s performance across three distinct layers of competence, which our diagnostic items (Q1-Q4) are designed to measure:

1. **Sociolinguistic Competence (The “Form”):** The knowledge of appropriate linguistic forms, grammar, and vocabulary for a given social context (e.g., *Keigo*).
2. **Socio-Cultural Competence (The “Values”):** The knowledge of underlying cultural values, norms, beliefs, and attitudes relevant to the situation (e.g., collectivism, attitude towards gender roles).
3. **Strategic/Pragmatic Competence (The “Action”):** The ability to *use* language to execute a specific, goal-oriented social action or strategy (e.g., ’humbly accepting advice,’ ’balancing team goals’).

This three-level framework, detailed in Table 2, allows for a precise, non-circular analysis of our “one-size-fits-all” (i.e., failure in Levels 2 & 3) versus “context-aware” (i.e., success across all 3 levels) hypothesis.

It is important to note that the mapping of questionnaire items (Q1–Q4) to these three communicative layers serves as a **theory-driven analytical lens** rather than a psychometrically validated construct. The purpose of this mapping is explanatory, allowing us to dissect the qualitative differences in model performance. While not a strict psychometric scale, the mapping shown in Table 2 remains empirically grounded in our experimental design. It reflects the specific competencies measured by the diagnostic questions tailored to each dimension’s unique scenario. For example, the PDI scenario’s focus on responding appropriately to senior advice necessitated questions measuring both Linguistic Form (Layer 1) and Social Strategy (Layer 3). This ensures our sub-score analysis framework directly addresses the cultural challenges posed.

### Ethics statement

The study involving human participants was reviewed and approved by the Institutional Review Board of Nara Institute of Science and Technology (Approval Number: 2024-I-42). Recruitment and data collection were conducted from February 1, 2025 to February 28, 2025. All participants were adults (18 years or older). Written informed consent was obtained from all participants via the crowdsourcing platform (participants clicked an agreement button before starting the survey). The survey was administered anonymously, and participants were free to withdraw at any time without penalty.

## Results

This section presents the findings from our quantitative evaluation framework. We first report the overall JWCAS scores and model hierarchy, followed by the diagnostic sub-score analysis used to explain these results.

### Questionnaire results

Table 3 reports the descriptive statistics for the holistic scores used in the JWCAS calculation, including the mean scores for each Hofstede dimension, standard deviations, 95% confidence intervals, and the final JWCAS score for each model.

**Table 3:** Mean scores, Standard Deviation (SD), 95% Confidence Interval (CI) and the final JWCAS score of each LLM on the six Hofstede dimensions based on crowdsourcing.

		PDI	IDV	UAI	MAS	LTO	IND	JWCAS
**LLM-jp**	Mean	3.60	3.42	3.28	3.60	3.38	3.32	3.43
	SD	0.80	0.89	0.88	0.80	0.77	0.80	
	95% CI	[3.50, 3.69]	[3.32, 3.53]	[3.18, 3.39]	[3.51, 3.69]	[3.29, 3.47]	[3.22, 3.41]	
**Llama**	Mean	3.38	3.26	3.40	3.37	3.29	3.25	3.33
	SD	0.85	0.91	0.85	0.87	0.85	0.79	
	95% CI	[3.28, 3.48]	[3.15, 3.36]	[3.30, 3.50]	[3.27, 3.47]	[3.19, 3.39]	[3.15, 3.34]	
**Phi**	Mean	3.68	3.57	3.44	3.57	3.33	3.43	**3.50**
	SD	0.85	0.83	0.83	0.86	0.86	0.82	
	95% CI	[3.59, 3.78]	[3.47, 3.66]	[3.34, 3.53]	[3.47, 3.66]	[3.23, 3.43]	[3.33, 3.53]	
**Qwen**	Mean	3.56	3.54	3.11	3.43	3.35	3.35	3.39
	SD	0.87	0.86	0.92	0.85	0.87	0.88	
	95% CI	[3.46, 3.66]	[3.44, 3.63]	[3.01, 3.22]	[3.34, 3.53]	[3.24, 3.45]	[3.25, 3.46]	
**GLM**	Mean	3.53	3.38	3.59	3.38	3.43	3.50	3.47
	SD	0.86	0.85	0.83	0.92	0.83	0.88	
	95% CI	[3.43, 3.63]	[3.28, 3.48]	[3.49, 3.69]	[3.27, 3.48]	[3.33, 3.53]	[3.40, 3.61]	

To statistically compare the models, we analyzed the holistic cultural alignment scores using a Linear Mixed-Effects Model (LMM). The analysis revealed a significant main effect of the model on the overall holistic cultural alignment scores (*F*(4, 8580) = 20.93, *p* < .001; $\chi^{2}(4)=83.70$, *p* < .001). It is noteworthy that the standard deviations (SDs) for the raw Likert-scale ratings were generally high, typically ranging from 0.8 to 0.9 across dimensions and models, indicating considerable variability in individual rater judgments.

Given this significant overall effect, we conducted pairwise post-hoc comparisons using Wald-type contrasts based on the LMM fixed-effect estimates. The resulting p-values were adjusted using the Benjamini–Hochberg False Discovery Rate (FDR) method to control for multiple pairwise comparisons. The results yielded a nuanced performance hierarchy with overlapping subsets:

- **Phi** (*M* = 3.50) emerged as the top-performing model, significantly outperforming the native *LLM-jp* (*M* = 3.43, $p_{fdr}=0.002$), *Qwen* (*M* = 3.39, $p_{fdr}<\mathrm{.001}$), and *Llama* (*M* = 3.32, $p_{fdr}<\mathrm{.001}$). It showed no statistically significant difference when compared to *GLM* (*M* = 3.47, $p_{fdr}=0.086$).
- **GLM** formed a highly competitive overlapping tier. It showed no significant difference from either *Phi* ($p_{fdr}=0.086$) or *LLM-jp* ($p_{fdr}=0.142$), while significantly outperforming both *Qwen* ($p_{fdr}=0.001$) and *Llama* ($p_{fdr}<\mathrm{.001}$).
- **LLM-jp** and **Qwen** formed the next overlapping subset, with no significant difference between them ($p_{fdr}=0.055$). While *LLM-jp* performed significantly worse than *Phi*, *Qwen* performed significantly worse than both *Phi* and *GLM*.
- **Llama** formed the bottom tier, scoring significantly lower than all other models (all $p_{fdr}\leq 0.002$).

**Fig 2:**
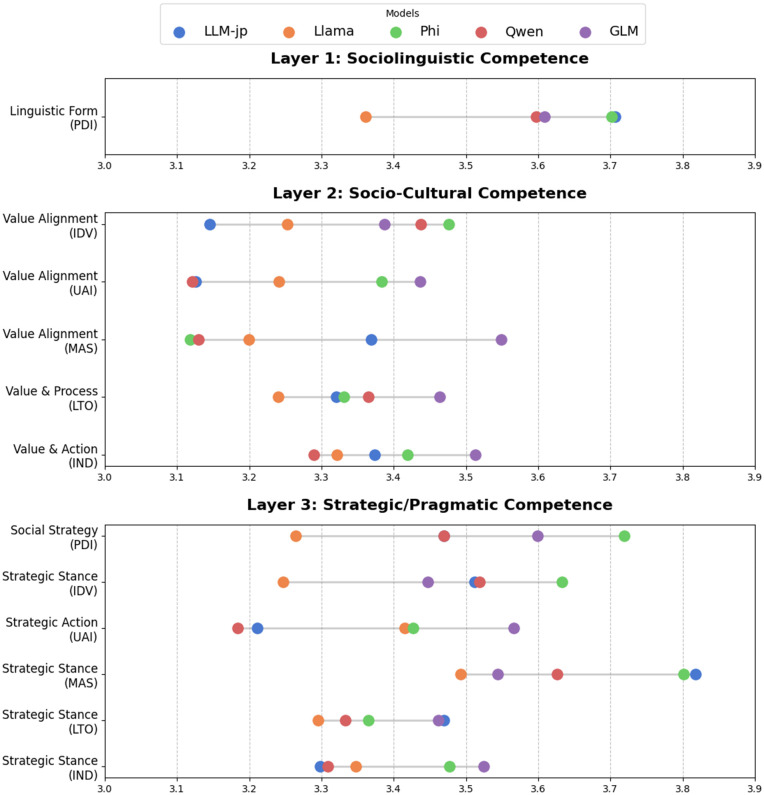
Diagnostic sub-score comparison across the three layers of communicative competence. This figure displays model performance (Mean Score 1-5) on the diagnostic categories belonging to each layer using dumbbell plots, arranged vertically by layer. The grey bar indicates the range (min to max score) for each category, while colored dots represent individual model scores. Note: To maintain visual clarity and avoid overlapping error bars among the five models, this plot visualizes the macro distributions. The precise Means, Standard Deviations (SDs), and 95% Confidence Intervals (CIs) for all diagnostic sub-scores plotted here are thoroughly detailed in S3 Table.

This nuanced performance hierarchy—particularly the finding that a multilingual model (Phi) significantly outperformed the native model, and another (GLM) achieved statistical parity with it—strongly motivates our subsequent layer-by-layer diagnostic analysis. To uncover the divergent underlying mechanisms driving these holistic scores, we analyzed the diagnostic sub-scores (Q1-Q4). Applying this framework as a theory-driven analytical lens, we mapped the items onto the Three-Layer Competence Model (see Table 2) and compared model performance across these layers. The findings reveal clear, systematic differences in model strategies—demonstrating how holistic scores can mask deep pragmatic disparities—visualized in the composite dumbbell plot shown in Fig 2. Detailed descriptive statistics for all diagnostic sub-scores are provided in S3 Table.

### Diagnostic sub-score results

#### Layer 1: sociolinguistic competence findings.

The *top panel* of Fig 2 presents the results for Layer 1, which consists of the “Linguistic Form (PDI)” category. Here, **LLM-jp** achieved the highest mean score (M = 3.706), closely followed by **Phi** (M = 3.702). **GLM** (M = 3.609) and **Qwen** (M = 3.597) performed slightly lower, while **Llama** scored the lowest (M = 3.361). This indicates strong performance in basic linguistic appropriateness, especially for the native model.

#### Layer 2: socio-cultural competence findings.

Performance on Layer 2 (Socio-Cultural Competence) is shown in the *middle panel* of Fig 2. Across the various value-related categories:

- **GLM** demonstrated consistently strong performance, achieving the highest or second-highest scores in “Value Alignment (UAI)” (M = 3.436), “Value & Process (LTO)” (M = 3.464), and “Value & Action (IND)” (M = 3.513). It also performed well in “Value Alignment (MAS)” (M = 3.549).
- **LLM-jp**, in contrast, showed significant weaknesses in this layer. While performing adequately in “Value & Action (IND)” (M = 3.374), it scored considerably lower in “Value Alignment (IDV)” (M = 3.145) and “Value Alignment (MAS)” (M = 3.369).
- **Phi** also performed well in this layer, particularly in “Value Alignment (IDV)” (M = 3.476) and “Value Alignment (UAI)” (M = 3.383).
- **Llama** and **Qwen** generally scored lower across most Layer 2 categories.

These results suggest significant differences in models’ abilities to grasp and reflect underlying cultural values.

#### Layer 3: strategic/pragmatic competence findings.

Finally, the *bottom panel* of Fig 2 illustrates performance on Layer 3 (Strategic/Pragmatic Competence). Key observations include:

- **GLM** again showed robust performance, notably scoring highest or second-highest in “Social Strategy (PDI)” (M = 3.599), “Strategic Stance (IDV)” (M = 3.447), “Strategic Action (UAI)” (M = 3.566), and “Strategic Stance (LTO)” (M = 3.462).
- **LLM-jp** exhibited a highly variable profile in this layer. It achieved the *highest* score among all models in “Strategic Stance (MAS)” (M = 3.818), demonstrating an ability to formulate a balanced statement in that context (e.g., appropriately balancing care for a colleague’s well-being with team performance goals). However, it performed relatively poorly in “Social Strategy (PDI)” (M = 3.469) and “Strategic Action (UAI)” (M = 3.211).
- **Phi** also showed strong performance, particularly in “Social Strategy (PDI)” (M = 3.719) and “Strategic Stance (IDV)” (M = 3.633).
- **Llama** consistently scored lowest across nearly all Layer 3 categories.

These findings highlight crucial differences in models’ abilities to execute culturally appropriate actions and strategies.

#### Summary of sub-score patterns.

Overall, the sub-score results presented in Fig 2 reveal distinct performance profiles that explain the holistic JWCAS results. **GLM** and **Phi** achieved their top-tier overall scores through consistent strength across all three layers of competence, particularly in executing appropriate socio-pragmatic strategies (Layer 3). In contrast, **LLM-jp** achieved a highly competitive overall score despite an imbalanced profile: it excelled in formal linguistic politeness (Layer 1) while showing weaknesses in aligning with specific cultural values (Layer 2) and executing adaptive strategies (Layer 3). **Llama** consistently underperformed across all layers. These patterns provide a mechanistic basis for understanding how different models navigate high-context cultural scenarios.

## Discussion

Our study reveals a complex relationship between holistic human evaluations and specific layers of cultural competence. At the overall evaluation level (JWCAS based on Q5), the leading multilingual models (**GLM** and **Phi**) achieved scores that rivaled or significantly surpassed the native model (**LLM-jp**). However, relying solely on this holistic metric obscures fundamental differences in how these models process and generate culturally embedded responses. Our layer-by-layer diagnostic analysis demonstrates that these competitive holistic scores are driven by markedly different underlying mechanisms.

### Explaining cultural alignment via the three-layer competence model

Our diagnostic sub-score analysis (Fig 2), based on the three-layer competence model (Table 2), reveals distinct strategic profiles that explain the overlapping high performance observed in the holistic JWCAS evaluations.

Despite achieving a top-tier holistic score, LLM-jp’s diagnostic profile suggests a “one-size-fits-all” Pragmatic Failure—defined as the inability to map surface-level linguistic utterances to their intended socio-cultural meanings [10]. While demonstrating strength in **Level 1 (Sociolinguistic Competence)**—mastering appropriate linguistic forms like *Keigo*—it appeared to falter consistently in **Level 2 (Socio-Cultural Competence)** (struggling to align with specific cultural values like collectivism or gender role nuances) and **Level 3 (Strategic/Pragmatic Competence)** (showing inconsistency in executing contextually appropriate social actions).

This highlights a focus on surface-level correctness over deep strategic execution, effectively “overfitting” to sociolinguistic forms. The fact that human evaluators still awarded LLM-jp high holistic scores suggests a strong anchoring effect: in high-context cultures, grammatical perfection can easily mask pragmatic inadequacies. This creates an illusion of fluency: the native model’s strong command of surface-level grammar may act as a linguistic veil, masking its weaknesses in executing the socio-pragmatic actions required in high-context Japanese workplace settings. To illustrate this concretely, consider the IDV (Individualism) “Performance Review Self-Eval” scenario from our dataset. A model suffering from this illusion might generate a self-evaluation using flawless formal Japanese (*Keigo*, satisfying Layer 1) but aggressively boast about personal achievements while disregarding the team’s mediocre results (failing the collectivist values of Layer 2 and the humble strategy of Layer 3). In contrast, models exhibiting context-aware strategies would use the same polite language to strategically emphasize shared team learning and mutual support, successfully balancing individual contributions with group harmony.

In contrast, the other top-tier models, GLM and Phi, achieved their high holistic scores by demonstrating balanced strength across all three layers. This raises a crucial question: *why* did they succeed in Layers 2 and 3, where the native model faltered? We hypothesize this difference is linked to the multilingual and multicultural diversity of their training data. GLM and Phi were trained or developed in broader multilingual settings, drawing on Chinese, English, and diverse web-scale data rather than only Japanese and English [16,19]. This broad exposure may have enabled them to learn more abstract, cross-cultural pragmatic concepts (e.g., “respect for hierarchy,” “maintenance of group harmony”) that are independent of their specific expression in any single language. This “abstract pragmatic transfer” would explain their “context-aware” strategy: they can flexibly apply these abstract concepts to the specific Japanese workplace scenarios. LLM-jp, conversely, learned *how* to speak politely but, due to a potential lack of diverse cultural data, may not have fully captured the abstract pragmatic rules governing *when* and *why* to use these forms (Layers 2 & 3).

Because we did not directly examine the models’ internal representations or training data composition, these explanations regarding abstract pragmatic transfer remain speculative. Alternative factors—such as differences in the diversity of Reinforcement Learning from Human Feedback (RLHF) datasets, specific instruction-tuning strategies, or broader cross-lingual alignment techniques—are equally plausible drivers of these performance gaps and warrant future investigation.

### Implications for LLM cultural competence

Our findings suggest that evaluating LLM cultural competence may require moving beyond holistic, single-metric evaluations. The three-layer framework offers a structured approach to diagnose specific socio-pragmatic weaknesses that are easily masked by surface-level linguistic proficiency—a phenomenon clearly observed in our native model’s results. This distinction appears critical for developing and deploying LLMs responsibly in real-world cross-cultural scenarios where pragmatic failures can lead to misunderstandings.

Furthermore, although the mean differences between the top-performing and lower-performing models appear relatively small numerically (e.g., a 0.5-point difference on a 5-point scale), these differences may have meaningful practical implications. In high-context Japanese workplaces, the boundary between “acceptable” and “offensive” is often subtle; a model lacking strategic pragmatics might generate a response that is grammatically flawless yet causes critical interpersonal friction.

### Limitations and future work

Several limitations should be acknowledged. First, while our study employed five diverse prompts per dimension to ensure robust evaluation, our analysis was limited to single-turn interactions. Future work should explore multi-turn dialogues to assess how models navigate evolving conversational dynamics and repair cultural misunderstandings in real-time.

Second, crowdsourced ratings, despite achieving good reliability (ICC = 0.837), might not fully capture expert nuances. The considerable variability observed in individual ratings (SDs 0.8–0.9) underscores the inherent subjectivity of evaluating cultural appropriateness and suggests that noise in the raw data might temper the precision of the findings, even with aggregate reliability being good. Future work could incorporate expert evaluations to validate these subtle pragmatic boundaries.

Third, our study relies heavily on Hofstede’s cultural dimensions. While highly useful as a quantifiable, heuristic framework to operationalize cross-cultural comparisons, this model has been criticized for potential cultural essentialism and treating national culture as static [5]. Future research could incorporate alternative cross-cultural frameworks or bottom-up, qualitative approaches to capture the fluidity of contemporary workplace norms.

Fourth, our item categorization, while theoretically grounded, could be complemented by direct computational linguistic analyses (e.g., exploring linguistic feature quantification) to automatically detect markers of pragmatic failure.

Finally, we acknowledge the rapid evolution of LLM architectures. The findings presented here serve as a temporal snapshot of model capabilities as of early 2025. Future iterations of these models will likely exhibit different cultural alignment profiles. Therefore, continuous evaluation and replication with newer, more advanced models remain crucial next steps for the research community.

## Conclusion

This study proposed and evaluated a framework combining the holistic JWCAS metric with a three-layer diagnostic sub-score analysis to assess LLM cultural alignment in typical Japanese workplace scenarios. Our results indicate that leading multilingual models (Phi and GLM) achieved overall cultural alignment scores that rivaled or significantly surpassed the native Japanese model (LLM-jp). Crucially, our three-layer framework exposed fundamental differences in their underlying communicative strategies. We hypothesize that the robust socio-pragmatic execution of GLM and Phi stems from their broader multilingual and multicultural training, which may enable abstract pragmatic transfer (competence in Layers 2 and 3). In contrast, LLM-jp effectively overfitted to linguistic forms (Layer 1), employing a rigid, “one-size-fits-all” strategy that is insufficient for complex, context-aware pragmatic execution.

Ultimately, our findings caution researchers and developers against evaluating cross-cultural AI alignment based solely on holistic metrics or linguistic proficiency. Such approaches risk creating an illusion of fluency, wherein models appear culturally aligned due to flawless native-level grammar, yet still fail to perform the socio-pragmatic actions required in real-world workplace communication. Developing truly culturally aware AI systems requires moving beyond single-metric evaluations and adopting multi-dimensional diagnostic frameworks that explicitly disentangle what a model says from the social action it intends to perform. Future work should aim to further validate the mechanisms of abstract pragmatic transfer and apply this multi-layered diagnostic approach to other high-context cultural environments.

## Supporting information

S1 Table Experimental prompts used in this study. Five scenarios were designed for each Hofstede dimension to assess model alignment across varied Japanese workplace contexts. Bold text indicates the descriptive title for each scenario.
(PDF)

S2 Table Questionnaire Items Used for Human Evaluation (English Translation). The following are the questionnaire items used in human evaluations and their English translations, provided for reader convenience. Participants rated LLM responses on a 5-point Likert scale (1: Strongly Disagree to 5: Strongly Agree) for each item corresponding to the specific scenario’s cultural dimension.
(PDF)

S3 Table Descriptive Statistics for Diagnostic Sub-scores. Mean scores, Standard Deviations (SDs), and 95% Confidence Intervals (CIs) for all five models across the diagnostic categories (Q1-Q4) used in the layer-by-layer analysis.
(PDF)

S4 Table Distribution of Human Evaluations. The number of valid crowdsourced evaluation sessions (total N = 1,718) distributed across the five LLMs and the six Hofstede cultural dimensions.
(PDF)
