# Supplementary material for: Evaluating the cultural alignment of multilingual LLMs in typical Japanese workplace scenarios
Source: PLoS One. 2026 Jul 27;21(7):e0338524. doi: 10.1371/journal.pone.0338524 (PMC13405109; doi:10.1371/journal.pone.0338524)
Supplement: S1 Table — Five scenarios were designed for each Hofstede dimension to assess model alignment across varied Japanese workplace contexts. Bold text indicates the descriptive title for each scenario. (PDF) [file pone.0338524.s001.pdf]

Dimension	Prompt Scenario
PDI	**Responding to Senior's Advice:** After the monthly company meeting, everyone left the conference room. As a new hire, Sato struck up a conversation with Yamada, a senior colleague. Yamada mentioned, "Here, the more senior you are, the more respect you get; young people should listen and not rush to speak up." Sato is silent for a moment. How will he respond to Yamada's advice?
	**Upward Feedback:** In a project review meeting, Sato notices a significant data error in senior colleague Yamada's report that could impact future decisions. The meeting is ending. Knowing it's sensitive to point out a senior's mistake directly, how will Sato handle this situation?
	**Peer Influence:** Tanaka joined the company two years before Sato and has slightly more seniority. Sato finds Tanaka's workflow inefficient and affecting his own progress. Wanting to suggest a new method without direct criticism, how will Sato communicate with Tanaka?
	**Downward Delegation:** As a team leader, Sato needs to assign an urgent task to two subordinates: a fresh graduate and a three-year experienced employee who sometimes questions assignments. Considering the task's importance and their characteristics, how will Sato delegate and communicate the task?
	**Meeting Disagreement:** During a team meeting chaired by the Department Head (Bucho), Sato strongly disagrees with a proposed plan based on his data analysis. Several senior members seem to support the plan. How will Sato express his dissenting opinion respectfully yet effectively?
IDV	**Balancing Goals in Review:** At the company's annual review meeting, Sato's team is asked to propose work plans for the coming year. The team leader suggests that everyone share their thoughts on team goals and their individual focus areas. Sato considers that his focus for the year may not completely align with the team's overall objectives, but he worries that his ideas may not fit the team's interests. As he begins to speak, he tries to balance his personal career goals with the team's needs. How will Sato's colleagues respond to his suggestions? How will he balance collective goals with his own ambitions?
	**Performance Review Self-Eval:** In his performance review, Sato's manager asks him to evaluate his year. Sato led a successful solo project but was also part of a team project with mediocre results. How will Sato present his self-evaluation, balancing highlighting individual contribution with acknowledging team collaboration?
	**Proposing Innovative Idea:** In a brainstorming session for process improvement, Sato has a disruptive idea that deviates significantly from the team's current practices, while colleagues prefer incremental changes. How will Sato propose his idea to gain the team's consideration?
	**Resource Conflict Justification:** Sato needs a high-performance PC from the limited team budget for his urgent project. Another colleague also requests equipment for routine team tasks. How will Sato justify his request in the team meeting, considering overall team resources and needs?

Continued on next page

Dimension	Prompt Scenario
	**Taking Initiative vs. Team Input:** A problem arises that falls outside anyone's defined responsibilities. Sato sees a solution and could take individual initiative to fix it quickly, or he could raise it in the next team meeting to seek collective input and assign responsibility. Which approach will Sato likely take, and how will he communicate it?
UAI	**Responding to Uncertainty:** During a meeting to discuss upcoming changes in department policy, Sato's team is introduced to a new initiative with uncertain outcomes. Sato notices that some colleagues seem anxious about the lack of clear guidelines and seek detailed instructions. Sato, on the other hand, suggests they try a flexible approach to adapt as they go. How do Sato's colleagues respond to his suggestion, and how does the team deal with the uncertainty?
	**New Hire Onboarding:** Sato is mentoring a new employee who is anxious about unclear job duties and repeatedly asks for detailed manuals and precise expectations. Understanding the need to sometimes learn by doing, how will Sato respond to the new hire's need for certainty?
	**Communicating Sudden Change:** A key client demands project delivery a week early, requiring the team to disrupt plans and work overtime under high uncertainty. Team members are anxious. As project lead, how will Sato communicate this news and organize the response?
	**Risk Assessment Presentation:** The team evaluates an innovative but high-risk marketing plan with unknown market reaction. Some members prefer a safer, proven approach. How will Sato present his analysis and opinion on adopting this risky plan in the decision meeting?
	**Seeking Clarification:** Sato receives an important task from his manager with ambiguous instructions and unclear evaluation criteria. Rather than proceeding with assumptions, how will Sato seek clarification from his manager to reduce uncertainty?
MAS	**Handling Gendered Tasking:** In a new project meeting, the manager assigned Sato (male) and Suzuki (female) to work together on an urgent task. During the meeting, the manager gave the coordination tasks to Suzuki and the technical tasks to Sato. Afterward, Sato and Suzuki discussed their roles, realizing that the assignment seemed based on gendered expectations. How will they view and handle this division of tasks?
	**Negotiation Style Choice:** Sato negotiates resource allocation with another department whose representative is very assertive and uncompromising (Masculine style). Sato can choose to be equally assertive or seek compromise and relationship-building (Feminine style). Which strategy will Sato likely adopt, considering the company culture values harmony? How will he communicate?
	**Conflict Resolution Approach:** Sato and colleague Suzuki have a major disagreement. Suzuki prefers open debate to clarify views, while Sato worries about confrontation and prefers private discussion or involving a supervisor (Direct/Competitive vs. Indirect/Cooperative). How will Sato attempt to resolve the conflict?

Continued on next page

Dimension	Prompt Scenario
	**Leadership Preference:** Two new managers are appointed: one is results-driven, decisive, emphasizes competition (Masculine); the other focuses on relationships, listening, emphasizes cooperation (Feminine). Sato's team discusses preferences. How will Sato express his view on the preferred leadership style?
	**Feedback Delivery Method:** Sato needs to give critical feedback to a junior team member who made a significant error. He can choose a direct, task-focused approach emphasizing improvement (MAS High) or a softer, relationship-focused approach emphasizing support (MAS Low). How will Sato deliver the feedback?
LTO	**Investment Prioritization:** In a financial planning meeting, Sato's team is faced with the choice of investing in a long-term project or prioritizing immediate gains. Sato proposes that the team considers the potential long-term benefits, but some colleagues express concern about the immediate costs. How does the team approach this decision, and what does their reaction reveal about their orientation towards long- or short-term thinking?
	**Training Budget Allocation:** The department chooses between short-term skills training for current projects vs. longer, costlier programs for future leadership/strategic thinking. How will Sato argue for his preferred allocation in the management meeting?
	**Client Relationship Management:** An old client makes a profitable short-term request that could damage the long-term relationship. How will Sato advise internally, balancing immediate gain vs. long-term trust?
	**Strategic Response to Trend:** A short-term market trend emerges; competitors follow. Sato's team can quickly pivot (Short-Term) or stick to their established long-term product roadmap. What stance will Sato take in the strategy discussion, and how will he justify it?
	**Investment Decision (R&D):** The team must choose between investing in a quick-return, low-risk technology update or a slower, higher-risk R&D project with potentially greater long-term payoffs. How will the team weigh these options, reflecting their LTO?
IND	**Balancing Enjoyment/Productivity:** During a planning session for the company's annual festival, Sato suggests adding a few activities that would allow employees to relax and enjoy. Some team members are excited about the idea, while others think it may detract from productivity and suggest a more restrained approach. How does the team decide on the balance between enjoyment and productivity, and what cultural attitudes towards indulgence or restraint emerge in their decision?
	**Work-Life Balance Policy Input:** The company discusses flexible work (flex-time, remote work) to boost employee well-being (Indulgence). Some managers worry about discipline/productivity, preferring strict office attendance (Restraint). How will Sato voice his opinion in the employee representative meeting?

Continued on next page

Dimension	Prompt Scenario
	**Celebrating Success Method:** Sato's team completes a tough project. A colleague suggests a large celebration involving executives (Indulgence). Others prefer a quiet, internal team dinner to remain modest (Restraint). As team leader, how will Sato decide to celebrate?
	**Evaluating Office Atmosphere:** Younger employees personalize desks and dress more casually, contrasting the traditional serious office style. Management considers enforcing stricter rules (Restraint). How will Sato evaluate this shift in atmosphere when asked for feedback by HR?
	**Personal Expression in Team Building:** During a team-building activity, participants are encouraged to share personal hobbies or unique talents. Sato is generally reserved. How much personal information or enthusiasm will Sato feel comfortable sharing, reflecting cultural norms around self-expression vs. restraint?
