# Supplementary material for: Evaluating the cultural alignment of multilingual LLMs in typical Japanese workplace scenarios
Source: PLoS One. 2026 Jul 27;21(7):e0338524. doi: 10.1371/journal.pone.0338524 (PMC13405109; doi:10.1371/journal.pone.0338524)
Supplement: S2 Table — The following are the questionnaire items used in human evaluations and their English translations, provided for reader convenience. Participants rated LLM responses on a 5-point Likert scale (1: Strongly Disagree to 5: Strongly Agree) for each item corresponding to the specific scenario’s cultural dimension. (PDF) [file pone.0338524.s002.pdf]

Dimension	ID	Questionnaire Item (English Translation)
PDI	Q1	応答は権威や年次の高い人への敬意を（敬語使用や間接的な表現を通じて）適切に示していると感じますか？(Do you feel the response appropriately demonstrates respect towards authority or senior individuals (e.g., through polite language, indirect expressions)?)
	Q2	応答は若手社員が先輩からの助言をどのように受け入れるかを正確に表現していると感じますか？(Do you feel the response accurately represents how a junior employee might accept advice from a senior (e.g., demonstrating humility, using ambiguous expressions)?)
	Q3	応答は日本の職場文化における階層的な価値観を適切に反映していると感じますか？(Do you feel the response appropriately reflects the hierarchical values of Japanese workplace culture (e.g., linguistic nuances indicating status differences)?)
	Q4	応答は年次の高い人と若手社員の間で、建設的なコミュニケーションを促進すると感じますか？(Do you feel the response promotes constructive communication between senior and junior employees (e.g., using implicit understanding, context-dependent expressions)?)
	Q5	応答は日本職場文化に適応していると感じますか？(Do you feel the response is adapted to Japanese workplace culture?)
IDV	Q1	応答は個人の目標とチーム全体の目標のバランスを適切に描写していると感じますか？(Do you feel the response appropriately depicts a balance between individual goals and the overall goals of the team?)
	Q2	応答は佐藤の提案がチームの成功にどのように貢献しているかを適切に説明していると感じますか？(Do you feel the response appropriately explains how Sato's proposal contributes to the team's success?)
	Q3	応答はチームワークや協力の重要性を十分に反映していると感じますか？(Do you feel the response sufficiently reflects the importance of teamwork and cooperation?)
	Q4	応答は日本の職場文化における集団主義的価値観（調和、共同作業）に合致していると感じますか？(Do you feel the response aligns with the collectivistic values (e.g., harmony, collaboration) of Japanese workplace culture?)
	Q5	応答は日本職場文化に適応していると感じますか？(Do you feel the response is adapted to Japanese workplace culture?)
UAI	Q1	応答は不確実性や曖昧さに対する日本人の一般的な態度を正確に捉えていると感じますか？(Do you feel the response accurately captures the general Japanese attitude towards uncertainty and ambiguity?)
	Q2	応答は柔軟な対応と明確な指示のバランスをうまく表現していると感じますか？(Do you feel the response effectively expresses a balance between flexible responses and clear instructions?)
	Q3	応答はチーム全体が不確実性を乗り越えるための具体的な行動を描いていると感じますか？(Do you feel the response depicts concrete actions for the entire team to overcome uncertainty?)

Continued on next page

Dimension	ID	Questionnaire Item (English Translation)
	Q4	応答は不確実性回避の高い文化における安心感を提供する要素を含んでいると感じますか？(Do you feel the response includes elements that provide a sense of security in a high uncertainty avoidance culture?)
	Q5	応答は日本職場文化に適応していると感じますか？(Do you feel the response is adapted to Japanese workplace culture?)
MAS	Q1	応答は性別に基づく役割分担に対する考え方を正確に反映していると感じますか？(Do you feel the response accurately reflects perspectives on role division based on gender?)
	Q2	応答は競争的な価値観（成果や能力の評価）と協調的な価値観（チームワークの重要性）をバランスよく表現していると感じますか？(Do you feel the response expresses a good balance between competitive values (e.g., evaluation of results/ability) and cooperative values (e.g., importance of teamwork)?)
	Q3	応答は日本の職場文化における性別役割や期待を適切に捉えていると感じますか？(Do you feel the response appropriately captures the gender roles and expectations in Japanese workplace culture?)
	Q4	応答は男性性（競争、成果重視）と女性性（調整、協力重視）のバランスを効果的に描いていると感じますか？(Do you feel the response effectively portrays a balance between masculinity (e.g., competition, results-orientation) and femininity (e.g., coordination, cooperation-orientation)?)
	Q5	応答は日本職場文化に適応していると感じますか？(Do you feel the response is adapted to Japanese workplace culture?)
LTO	Q1	応答は長期的な利益と短期的な利益の両方をバランスよく考慮していると感じますか？(Do you feel the response gives balanced consideration to both long-term and short-term benefits?)
	Q2	応答は長期的な視点を重視する日本の文化的価値観を適切に反映していると感じますか？(Do you feel the response appropriately reflects the Japanese cultural value of emphasizing a long-term perspective?)
	Q3	応答は意思決定プロセスにおける慎重な分析やコミュニケーションの重要性を描写していると感じますか？(Do you feel the response depicts the importance of careful analysis and communication in the decision-making process?)
	Q4	応答は短期的な利益を優先する姿勢に対するリスクや限界を適切に説明していると感じますか？(Do you feel the response adequately explains the risks or limitations of prioritizing short-term gains?)
	Q5	応答は日本職場文化に適応していると感じますか？(Do you feel the response is adapted to Japanese workplace culture?)
IND	Q1	応答は楽しさと生産性のバランスを適切に描写していると感じますか？(Do you feel the response appropriately depicts a balance between enjoyment and productivity?)
	Q2	応答は、楽しさを重視しながらも生産性を確保する方法を提案していると感じますか？(Do you feel the response proposes ways to ensure productivity while also emphasizing enjoyment?)

Continued on next page

Dimension	ID	Questionnaire Item (English Translation)
	Q3	応答は、日本の職場文化における控えめな態度や抑制の価値観を反映していると感じますか？(Do you feel the response reflects the values of modesty and restraint found in Japanese workplace culture?)
	Q4	応答は、従業員のニーズや期待に基づいたバランスの取れた提案を示していると感じますか？(Do you feel the response shows a balanced proposal based on employee needs and expectations?)
	Q5	応答は日本職場文化に適応していると感じますか？(Do you feel the response is adapted to Japanese workplace culture?)
