# Supplementary material for: Evaluating the cultural alignment of multilingual LLMs in typical Japanese workplace scenarios
Source: PLoS One. 2026 Jul 27;21(7):e0338524. doi: 10.1371/journal.pone.0338524 (PMC13405109; doi:10.1371/journal.pone.0338524)
Supplement: S3 Table — Mean scores, Standard Deviations (SDs), and 95% Confidence Intervals (CIs) for all five models across the diagnostic categories (Q1-Q4) used in the layer-by-layer analysis. (PDF) [file pone.0338524.s003.pdf]

		LLM-jp	Llama	Phi	Qwen	GLM
Linguistic Form (PDI)	Mean	3.71	3.36	3.70	3.60	3.61
	SD	0.81	0.85	0.84	0.88	0.80
	95% CI	[3.64, 3.77]	[3.29, 3.43]	[3.63, 3.77]	[3.53, 3.67]	[3.54, 3.67]
Value Alignment (IDV)	Mean	3.15	3.25	3.48	3.44	3.39
	SD	0.98	0.96	0.91	0.90	0.88
	95% CI	[3.07, 3.22]	[3.17, 3.33]	[3.40, 3.55]	[3.36, 3.51]	[3.32, 3.46]
Value Alignment (UAI)	Mean	3.13	3.24	3.38	3.12	3.44
	SD	0.91	0.88	0.85	0.90	0.89
	95% CI	[3.05, 3.20]	[3.17, 3.31]	[3.31, 3.45]	[3.05, 3.19]	[3.36, 3.51]
Value Alignment (MAS)	Mean	3.37	3.20	3.12	3.13	3.55
	SD	0.95	0.92	0.97	0.95	0.98
	95% CI	[3.29, 3.45]	[3.12, 3.27]	[3.04, 3.20]	[3.05, 3.21]	[3.47, 3.63]
Value & Process (LTO)	Mean	3.32	3.24	3.33	3.37	3.46
	SD	0.78	0.82	0.80	0.81	0.82
	95% CI	[3.25, 3.39]	[3.17, 3.31]	[3.26, 3.40]	[3.30, 3.43]	[3.40, 3.53]
Value & Action (IND)	Mean	3.37	3.32	3.42	3.29	3.51
	SD	0.84	0.82	0.84	0.81	0.89
	95% CI	[3.30, 3.44]	[3.25, 3.39]	[3.35, 3.49]	[3.22, 3.36]	[3.44, 3.59]
Social Strategy (PDI)	Mean	3.47	3.26	3.72	3.47	3.60
	SD	0.84	0.89	0.88	0.92	0.86
	95% CI	[3.40, 3.54]	[3.19, 3.34]	[3.65, 3.79]	[3.39, 3.54]	[3.53, 3.67]
Strategic Stance (IDV)	Mean	3.51	3.25	3.63	3.52	3.45
	SD	0.85	0.95	0.85	0.85	0.86
	95% CI	[3.44, 3.58]	[3.17, 3.32]	[3.56, 3.70]	[3.45, 3.59]	[3.38, 3.52]
Strategic Stance (MAS)	Mean	3.82	3.49	3.80	3.63	3.54
	SD	0.82	0.89	0.92	0.86	0.92
	95% CI	[3.75, 3.88]	[3.42, 3.56]	[3.73, 3.88]	[3.56, 3.70]	[3.47, 3.62]
Strategic Stance (LTO)	Mean	3.47	3.29	3.37	3.33	3.46
	SD	0.78	0.82	0.81	0.79	0.83
	95% CI	[3.40, 3.53]	[3.23, 3.36]	[3.30, 3.43]	[3.27, 3.40]	[3.39, 3.53]
Strategic Stance (IND)	Mean	3.30	3.35	3.48	3.31	3.53
	SD	0.81	0.80	0.83	0.79	0.87
	95% CI	[3.23, 3.37]	[3.28, 3.41]	[3.41, 3.55]	[3.24, 3.37]	[3.45, 3.60]
Strategic Action (UAI)	Mean	3.21	3.41	3.43	3.18	3.57
	SD	0.91	0.86	0.87	0.86	0.89
	95% CI	[3.14, 3.29]	[3.34, 3.49]	[3.36, 3.50]	[3.11, 3.26]	[3.49, 3.64]
