# Supplementary material for: Evaluating the cultural alignment of multilingual LLMs in typical Japanese workplace scenarios
Source: PLoS One. 2026 Jul 27;21(7):e0338524. doi: 10.1371/journal.pone.0338524 (PMC13405109; doi:10.1371/journal.pone.0338524)
Supplement: S4 Table — The number of valid crowdsourced evaluation sessions (total N = 1,718) distributed across the five LLMs and the six Hofstede cultural dimensions. (PDF) [file pone.0338524.s004.pdf]

Dimension	No. of Evaluator Sessions	Models Evaluated per Session	Total Rating Data Points
**PDI**	294	All 5 models	7350
**IDV**	293	All 5 models	7325
**UAI**	282	All 5 models	7050
**MAS**	297	All 5 models	7425
**LTO**	275	All 5 models	6875
**IND**	277	All 5 models	6925
**Total**	1718	-	42950
