# Supplementary material for: Evaluating the cultural alignment of multilingual LLMs in typical Japanese workplace scenarios
Source: PLoS One. 2026 Jul 27;21(7):e0338524. doi: 10.1371/journal.pone.0338524 (PMC13405109; doi:10.1371/journal.pone.0338524)
Supplement: Attachment — Submitted filename: Reviewer Comments on Revised Manuscript.docx [file pone.0338524.s007.docx]

**Reviewer Comments on Revised Manuscript**

**Manuscript Title:** Evaluating the Cultural Alignment of Multilingual LLMs in Typical Japanese Workplace Scenarios
**Manuscript Number:** PONE-D-25-61951R1

Dear Authors,

Thank you for the opportunity to review the revised version of this manuscript. The revision is substantial and meaningful. Compared with the previous version, the manuscript is clearer in its conceptual framing, more transparent in its methods, and more cautious in the interpretation of its findings.

The authors have, in my assessment, addressed most of the major concerns raised in the first round. In particular, I commend the following improvements:

1. The authors clearly acknowledge and correct the earlier scoring inconsistency, and the Japanese Workplace Cultural Alignment Score (JWCAS) is now aligned with the stated methodology by being based on Q5 only.
2. The sampling and evaluation pipeline is now much better described, including the number of scenarios, generated responses, and valid human evaluation sessions.
3. The mapping from questionnaire items to the three-layer framework is now appropriately framed as a theory-driven analytical lens rather than a psychometrically validated measurement structure.
4. The explanation involving “abstract pragmatic transfer” is now presented more appropriately as a hypothesis, with alternative explanations also acknowledged.
5. The manuscript now includes a substantially improved limitations section, along with a public data/code repository.

These revisions materially improve the rigor and credibility of the manuscript.

That said, I still recommend **minor revision** before final acceptance, for the following reasons.

First, the statistical reporting would benefit from additional clarification. The manuscript mentions Tukey’s HSD for post-hoc testing and also states that Benjamini-Hochberg FDR correction was applied. The exact analytical workflow should be stated more explicitly so that readers can understand how multiplicity control was implemented. In addition, because the analysis is based on 5-point Likert-scale ratings modeled with an LMM, a brief justification for treating the outcome as approximately continuous would strengthen the methodological transparency.

Second, there is a residual inconsistency in the description of the evaluation procedure. The manuscript states that each participant saw “a single, anonymized LLM-generated response,” yet elsewhere states that each session involved evaluating outputs from all five models. This is likely resolvable through clearer wording, but the current phrasing may confuse readers regarding the unit of analysis and the session structure.

Third, the data availability statement is much improved and appears broadly consistent with PLOS ONE’s policy. However, I encourage the authors to provide a more stable archival form of the shared materials, such as a DOI-backed repository snapshot, or at least to specify the repository version or commit hash for reproducibility.

Fourth, the manuscript is generally intelligible and written in acceptable academic English, but minor copyediting is still needed. For example, the sentence stating that “these competitive holistic scores is driven” contains a grammatical error and should be corrected. A final proofreading pass is recommended.

In summary, the manuscript is now close to publishable form. The main substantive concerns from the prior review round have been satisfactorily addressed. My remaining comments are minor and pertain mainly to reporting precision, wording clarity, and final language cleanup. I would support publication after these final issues are resolved.

Sincerely,
Reviewer
