# Supplementary material for: Evaluating the cultural alignment of multilingual LLMs in typical Japanese workplace scenarios
Source: PLoS One. 2026 Jul 27;21(7):e0338524. doi: 10.1371/journal.pone.0338524 (PMC13405109; doi:10.1371/journal.pone.0338524)
Supplement: Attachment — Submitted filename: Final Comments.docx [file pone.0338524.s005.docx]

1. There is suggestion that the authors may revise the title to improve clarity and conciseness. In particular, the authors are encouraged to consider **“Japanese Workplace Cultural Adaptability in Multilingual Language Models”** or **“Cultural Sensitivity of Multilingual LLMs in Japanese Work Contexts”** as alternative titles.
2. The abstract does not report essential methodological details, including the use of crowdsourced human evaluators, approximate sample size, and study limitations, which should be added for transparency.
   The research gap is not explicitly articulated and should be clearly positioned in relation to existing studies on cultural bias and cultural alignment in LLMs.
3. The manuscript lacks a concise and structured statement of its key contributions, which should be added.
4. Well-documented critiques of Hofstede’s model, including cultural essentialism and contextual rigidity, are not addressed and should be acknowledged.
5. The sampling and evaluation pipeline requires clearer explanation, particularly regarding the transition from generated model outputs to the final set of human-evaluated responses.
6. The relatively small mean differences between models are not sufficiently contextualized in terms of their real-world relevance to workplace communication.
7. Interpretations related to training data diversity, model overfitting, and “abstract pragmatic transfer” are speculative and not empirically tested, and should therefore be framed more cautiously as hypotheses.
8. The manuscript does not address the temporal validity of the findings despite the rapid evolution of LLM architectures.
9. Greater inclusion of peer-reviewed literature from cross-cultural communication, organizational behavior, and AI ethics is required to strengthen the scholarly foundation.
10. The manuscript requires clearer articulation of its contributions, stronger theoretical reflexivity, improved methodological transparency, more cautious interpretation of results, and broader engagement with peer-reviewed literature before it can be considered for publication.
