# Supplementary material for: Evaluating the cultural alignment of multilingual LLMs in typical Japanese workplace scenarios
Source: PLoS One. 2026 Jul 27;21(7):e0338524. doi: 10.1371/journal.pone.0338524 (PMC13405109; doi:10.1371/journal.pone.0338524)
Supplement: Attachment — Submitted filename: response_letter.docx [file pone.0338524.s006.docx]

Mar 13, 2026

Dear Dr. Hassan,

Thank you for giving us the opportunity to submit a revised draft of our manuscript. We deeply appreciate the time and effort that you and the reviewers have dedicated to evaluating our work. The constructive feedback has been instrumental in significantly improving the rigor, clarity, and depth of our paper.

We have carefully considered all comments and revised the manuscript accordingly. Below, we provide a point-by-point response to each reviewer's comments.

**General Response: Clarification on the Updated Statistical Data**

Before addressing the specific comments, we would like to transparently report a correction made to the statistical results in this revised manuscript.

During the revision process, we discovered a transcription oversight in our initial submission. The original Japanese Workplace Cultural Alignment Score (JWCAS) values reported in the text were inadvertently pulled from a preliminary calculation that averaged all questions (Q1–Q5), rather than the strictly defined holistic metric (Q5 only) as stated in our methodology.

To rectify this, we have corrected the JWCAS calculations to exclusively reflect Q5. Consequently, we have also updated the corresponding statistical analysis throughout the **Results** section, employing a rigorous Linear Mixed-Effects Model (LMM) with FDR correction to ensure total accuracy and methodological alignment.

Importantly, while the specific numerical values have changed, this robust re-analysis has strengthened our core findings. The updated results explicitly confirm that the leading multilingual models (Phi and GLM) achieved overall cultural alignment scores that rivaled or even significantly surpassed the native Japanese model (LLM-jp). This provides even stronger empirical support for our hypotheses regarding the "Illusion of Fluency" and "Abstract Pragmatic Transfer" discussed in the paper.

To ensure complete transparency and to support the *PLOS ONE* data policy, we have open-sourced our entire analytical pipeline. All cleaned human evaluation data and code containing our statistical calculations are now publicly available in our repository: <https://github.com/sociocom/cultural_alignment_japanese_workplace>

**Point-by-Point Responses to Reviewers**

**Reviewer #1**

**Comment 1.1:** *While the integration of Hofstede’s dimensions with a three-layer communicative competence model is innovative, the mapping of questionnaire items (Q1–Q4) to specific layers (Table 2) appears to be post-hoc and lacks empirical validation... Provide a clear, principled justification... or explicitly acknowledge the mapping as a theory-driven analytical lens. Briefly acknowledge known critiques of Hofstede’s model.*

**Response:** We thank the reviewer for this excellent point. We completely agree that the mapping is explanatory rather than psychometrically validated. As suggested, we have explicitly reframed this mapping as a "theory-driven analytical lens" used to dissect qualitative differences in model performance, rather than an absolute psychometric construct. Furthermore, we have added explicit acknowledgments of the critiques of Hofstede’s model (e.g., cultural essentialism, static monoliths) in both the **Introduction** and **Limitations** sections.

**Comment 1.2:** *The paper’s key interpretive claim—that GLM/Phi’s superior performance stems from “abstract pragmatic transfer”... remains hypothetical. No evidence is presented regarding training data composition... Reframe this explanation as a plausible hypothesis.*

**Response:** We appreciate this vital methodological critique. We have revised the manuscript to strictly frame "abstract pragmatic transfer" as a hypothesis rather than a proven conclusion. In the **Discussion** section, we have also added a paragraph discussing alternative factors that could drive these performance gaps, such as differences in Reinforcement Learning from Human Feedback (RLHF) datasets and specific instruction-tuning strategies.

**Comment 1.3:** *The reported standard deviations (0.8–0.9 on a 5-point scale) indicate substantial disagreement among raters... Perform a post-hoc analysis of rater disagreement... Discuss how demographic skew (age, possibly industry/role) might influence ratings.*

**Response:** We thank the reviewer for this perceptive comment. To address the request for a breakdown of rater variability, we point to our newly added **S3 Table** to the Supporting Information. This table details the Standard Deviations (SDs) across all specific models and diagnostic dimensions. The analysis reveals that high variability (SDs generally between 0.8 and 1.0) is a consistent feature across almost all models and cultural dimensions, indicating that this disagreement is likely an inherent characteristic of evaluating subjective cultural norms rather than an anomaly specific to one model or prompt. Furthermore, we have explicitly discussed this high variability, along with the demographic skew of our crowdsourced sample (which leans toward experienced, older professionals), in the **Limitations** section, noting how it might bias interpretations toward more traditional workplace norms.

**Comment 1.4:** *The selection of the 1,718 evaluations from a larger pool is insufficiently described. The criteria for “representative sampling” are unclear... Clearly detail the sampling procedure.*

**Response:** We apologize for the lack of clarity. We have expanded the **Methods** section to explicitly detail the stratified random sampling procedure. We clarified that a total of 30,000 texts were generated (1,000 per model per dimension), from which responses were dynamically and randomly sampled for human evaluators, yielding the final 1,718 valid sessions. Furthermore, exactly as requested, we have added **S4 Table** to the Supporting Information, which clearly reports the distribution of these 1,718 evaluations across all models and Hofstede dimensions.

**Comment 1.5:** *Include 1–2 example prompts and model responses in the main text... Replace min–max bars with 95% confidence intervals... Table 3: Add a column for standard deviations... Briefly discuss the real-world consequences... Define key terms... Ensure acronyms are spelled out.*

**Response:** We sincerely thank the reviewer for these excellent suggestions, which have greatly enhanced the readability and precision of our manuscript. We have addressed all of these points as follows:

1. **Example Prompts:** We have integrated a detailed descriptive example of a PDI scenario (correcting a superior's mistake) directly into the **Methods** section (Page 4, Lines 105-113) to illustrate the divergence between grammatical politeness and pragmatic strategy.
2. **Table 3 (SDs):** We have added the Standard Deviations (SDs) alongside the means and CIs for the holistic scores (Q5) in Table 3, as requested.
3. **Figure 2 (CIs):** Figure 2 visualizes the diagnostic sub-scores (Q1-Q4). We encountered a visualization challenge here: plotting five overlapping 95% CIs on a single horizontal axis for every sub-score category rendered the dumbbell plot highly cluttered and unreadable. To ensure visual clarity while strictly adhering to your request for statistical rigor, we retained the current plot format to illustrate macro-trends. However, we have calculated the precise Means, SDs, and 95% CIs for all diagnostic sub-scores (Q1-Q4) and compiled them into a new supplementary table (**S3 Table**). We have added a note in the Figure 2 caption directing readers to this table for the complete statistical intervals.
4. **Real-world Consequences:** We have expanded the **Implications** subsection to explicitly discuss how pragmatic failures (despite flawless grammar) can cause critical interpersonal friction in high-context environments.
5. **Definitions & Acronyms:** We have defined "Pragmatic Failure" upon its first use in both the Abstract and the Discussion, and we have ensured all Hofstede dimension acronyms are spelled out upon their initial introduction.

**Reviewer #2**

*(Note to authors: Several points raised by Reviewer 2 appear to reference experimental parameters that differ significantly from our actual methodology. We respectfully clarify our actual study design below.)*

**Comment 2.1:** *The manuscript does not sufficiently explain why Japanese workplace culture was selected over other non-Western cultures (e.g., Chinese, Indian). I recommend that the authors strengthen the justification for this specific cultural focus in the Introduction section.*

**Response:** We thank the reviewer for this insightful recommendation. We have expanded the **Introduction** section to explicitly justify our focus on the Japanese workplace. We clarified that Japan serves as a uniquely rigorous "stress-test" environment for LLMs. Unlike many other non-Western cultures, Japanese workplace communication features a highly codified, rigid socio-linguistic layer (Honorifics / *Keigo*) that frequently diverges from the underlying pragmatic intent (the high-context necessity of "reading the air" or *Kuuki wo yomu*). This unique dichotomy makes it the perfect cultural environment to test our core hypothesis: distinguishing between an LLM's surface-level "Illusion of Fluency" and its true, deep socio-pragmatic alignment.

**Comment 2.2:** *Insufficient prompt design details... Single sampling issue: Each scenario was sampled only once (single run)... Limited number of evaluation scenarios: Only seven virtual scenarios were used.*

**Response:** We respectfully wish to clarify the scale of our study design, which may have been misunderstood in our initial description. Our study did not use a single run or only seven scenarios. As now detailed explicitly in our revised **Methods** section, we constructed **30 distinct evaluation scenarios** (5 unique scenarios for each of the 6 Hofstede dimensions). Furthermore, each model generated 1,000 responses per dimension, resulting in a total corpus of **30,000 generated responses**, mitigating the randomness of single-run outputs. To address the request for prompt details, we have also added a descriptive example of a PDI scenario directly into the main text, and the complete English translations of all 30 prompt templates are provided in **S1 Table**.

**Comment 2.3:** *Evaluator Sampling: Only three evaluators were used, which is a relatively small sample... The reliability for cultural appropriateness (0.61) is lower...*

**Response:** We believe there may be a critical misunderstanding regarding our evaluator pool. Our study utilized a large-scale crowdsourcing approach, yielding **1,718 valid human evaluation sessions**, not three evaluators. Because each session evaluated the outputs of all five models across the five questionnaire items, this generated a massive dataset of **42,950 individual Likert-scale rating data points**. To make this completely transparent, we have added **S4 Table**, which maps the distribution of these 1,718 sessions across the cultural dimensions. Additionally, our calculated Intraclass Correlation Coefficient (ICC) for the evaluations is **0.837** (indicating excellent reliability), not 0.61. We have ensured these methodological details are maximally prominent in the revised **Methods** section.

**Comment 2.4:** *Consider introducing more contemporary evaluation frameworks... Employ the “LLM-as-a-judge” approach as a supplementary evaluation method. Adopt multi-turn dialogue evaluation protocols.*

**Response:** We deeply appreciate these forward-looking methodological suggestions. While "LLM-as-a-judge" is highly efficient for general instruction-following benchmarks, we deliberately avoided it for this specific study. Recent literature highlights that state-of-the-art LLMs exhibit strong Western cultural biases. Using a potentially culturally biased LLM to evaluate the nuanced, high-context socio-pragmatic behaviors of other models risks circular validation and could mask subtle cultural misalignments. Therefore, we maintained our crowdsourced human evaluation, as native human judgment remains the absolute gold standard for assessing authentic cultural resonance. Regarding multi-turn dialogue, we completely agree it represents the next frontier for cultural evaluation, and we have explicitly added this to our **Limitations and Future Work** section.

**Comment 2.5:** *Discussion lacks depth... Unclear theoretical contributions... Superficial limitations discussion.*

**Response:** We thank the reviewer for pushing us to deepen our scholarly discussion. In the revised manuscript, we have significantly expanded the **Discussion** and **Implications** sections. We clearly articulate our theoretical contribution: moving beyond single-metric evaluations to a multi-layered diagnostic approach (differentiating Form, Values, and Action). We also expanded the **Limitations** section to thoroughly discuss the subjectivity and demographic skew of human evaluations, the temporality of LLM architectures, and the critiques of Hofstede's model (e.g., cultural essentialism).

**Reviewer #3**

**Comment 3.1:** *There is a suggestion that the authors may revise the title to improve clarity and conciseness. In particular, the authors are encouraged to consider “Japanese Workplace Cultural Adaptability in Multilingual Language Models” or “Cultural Sensitivity of Multilingual LLMs in Japanese Work Contexts” as alternative titles.*

**Response:** We sincerely thank the reviewer for these excellent suggestions. We entirely agree that a more concise title strengthens the paper. We have revised the title to: **"Evaluating the Cultural Alignment of Multilingual LLMs in Typical Japanese Workplace Scenarios"**, which captures the essence of both of your suggestions while maintaining precision.

**Comments 3.2, 3.3 & 3.10:** *The abstract does not report essential methodological details... The research gap is not explicitly articulated... The manuscript lacks a concise and structured statement of its key contributions.*

**Response:** We deeply appreciate this structural feedback. We have extensively overhauled the **Abstract** and the final paragraphs of the **Introduction** to explicitly address these omissions:

1. **Research Gap:** We now explicitly state the gap: while existing evaluations predominantly focus on Western contexts or rely on static multiple-choice benchmarks, our study addresses the critical need to evaluate generative, high-context non-Western socio-pragmatic alignment.
2. **Methodological Details:** We have incorporated the essential figures into the Abstract, specifying the use of crowdsourced human evaluators, the sample size (1,718 valid sessions generating 42,950 rating data points), and the dual-evaluation framework.
3. **Key Contributions:** We have added a concise statement in both the Abstract and Introduction highlighting our main contribution: demonstrating that holistic evaluation metrics can obscure deep pragmatic deficits (the "Illusion of Fluency"), and that our layer-by-layer diagnosis reveals the distinct strategies models use to achieve alignment.

**Comments 3.4 & 3.9:** *Well-documented critiques of Hofstede’s model (cultural essentialism) are not addressed... Greater inclusion of peer-reviewed literature from cross-cultural communication is required.*

**Response:** This is a crucial theoretical point. We have explicitly acknowledged the limitations of Hofstede's framework (specifically cultural essentialism and the risk of treating national cultures as static monoliths) in the **Introduction** and **Limitations** sections. Furthermore, to strengthen our theoretical reflexivity, we have integrated foundational cross-cultural and pragmatic literature (e.g., Hall, 1976; Meyer, 2014; Ide, 1989; Thomas, 1983). These additions help justify our selection of the Japanese workplace not merely as an application case, but as a uniquely rigorous "stress-test" environment due to its extreme high-context nature and rigid honorific system.

**Comment 3.5:** *The sampling and evaluation pipeline requires clearer explanation, particularly regarding the transition from generated model outputs to the final set of human-evaluated responses.*

**Response:** We apologize for the previous lack of clarity. We have significantly expanded the **Methods** section to explicitly detail the stratified random sampling pipeline. We clarified the transition: 30,000 texts were initially generated (1,000 per model per dimension), from which responses were dynamically and randomly sampled for human evaluators. To provide ultimate transparency, we have also added **S4 Table**, which explicitly maps the distribution of the 1,718 human-evaluation sessions and the 42,950 rating data points across the different cultural dimensions.

**Comment 3.6:** *The relatively small mean differences between models are not sufficiently contextualized in terms of their real-world relevance to workplace communication.*

**Response:** We completely agree. We have added a dedicated paragraph in the **Implications for LLM cultural competence** section to contextualize this. We explain that in high-context environments like the Japanese workplace, the boundary between "acceptable" and "offensive" is incredibly subtle. Even a numerically small difference on a 5-point scale can represent the critical threshold between maintaining professional harmony and causing severe interpersonal friction (i.e., pragmatic failure despite flawless grammar).

**Comments 3.7 & 3.8:** *Interpretations related to "abstract pragmatic transfer" are speculative and not empirically tested, and should therefore be framed more cautiously as hypotheses. The manuscript does not address the temporal validity of the findings.*

**Response:** We thank the reviewer for guiding us to state our analytical boundaries more rigorously. We have systematically toned down the causal language throughout the revised manuscript. In the **Discussion** and **Conclusion** sections, "abstract pragmatic transfer" is now strictly framed as a *proposed hypothesis*. Furthermore, we have explicitly incorporated alternative explanatory mechanisms (such as differences in parameter scaling, the diversity of RLHF datasets, and specific instruction-tuning strategies) as equally plausible drivers. Finally, we have added a new paragraph in the **Limitations** section explicitly acknowledging the rapid, temporal evolution of LLM architectures, noting that our findings represent a snapshot of model capabilities as of early 2025.

We hope that these revisions and clarifications fully address the concerns raised by the reviewers and that the manuscript is now suitable for publication in *PLOS ONE*.

Thank you once again for your constructive guidance.

Sincerely,

Eiji Aramaki, PhD

Nara Institute of Science and Technology

8916-5 Takayama-cho, Ikoma, Nara 630-0192, JAPAN (On behalf of all authors)
