# Supplementary material for: Evaluating the cultural alignment of multilingual LLMs in typical Japanese workplace scenarios
Source: PLoS One. 2026 Jul 27;21(7):e0338524. doi: 10.1371/journal.pone.0338524 (PMC13405109; doi:10.1371/journal.pone.0338524)
Supplement: Attachment — Submitted filename: response_letter_2.docx [file pone.0338524.s008.docx]

May 29, 2026

Dear Academic Editor and Reviewers,

We sincerely thank the Academic Editor and the reviewers for their careful evaluation of our revised manuscript. We are grateful that the reviewers found the manuscript substantially improved and technically sound. In this revision, we have addressed the remaining minor comments by clarifying the statistical reporting, resolving the wording inconsistency in the evaluation procedure, strengthening the reproducibility information in the Data Availability Statement, and conducting a final language-polishing pass.

Below, we provide a point-by-point response to the remaining comments.

**Response to the Academic Editor**

**Comment:** *The reviewer is satisfied with the substantial improvements and considers the manuscript technically sound. However, before a final decision can be made, please address the remaining minor comments raised by the reviewer carefully. In particular, please clarify the statistical reporting, resolve the wording inconsistency in the evaluation procedure, strengthen the reproducibility details in the data availability statement, and complete a final language-polishing pass.*

**Response:** We thank the Academic Editor for the positive assessment and for summarizing the remaining issues. In the revised manuscript, we clarified the statistical reporting, corrected the description of the evaluation procedure, updated the Data Availability Statement in the submission system with version-specific reproducibility information, and conducted a final language-polishing pass. We have also revised the manuscript text and reference list where needed.

**Response to Reviewer #1**

**Comment:** *All comments have been addressed. Thank you for your careful and thorough responses to my comments. The revisions are appreciated and have strengthened the paper. I have no further questions.*

**Response:** We sincerely thank the reviewer for the positive evaluation and for the helpful comments in the previous round. We are grateful that the reviewer found the revisions satisfactory.

**Response to Reviewer #2**

**Comment 1:** *The statistical reporting should be made more explicit. The manuscript states that post-hoc comparisons were conducted using Tukey’s HSD, but it also states that Benjamini-Hochberg FDR correction was applied. Please clarify exactly how these procedures were combined. In addition, because the outcome consists of 5-point Likert ratings analyzed with an LMM, it would be helpful to briefly justify the treatment of these ratings as approximately continuous, or at least acknowledge this modeling choice as a pragmatic approximation.*

**Response:** We thank the reviewer for pointing out this ambiguity. We have clarified the statistical analysis in the revised manuscript. Specifically, the post-hoc analysis did not combine Tukey’s HSD with Benjamini–Hochberg correction. The earlier wording was imprecise, and we have revised it throughout the manuscript.

In the final analysis, after fitting the Linear Mixed-Effects Model (LMM), we conducted pairwise model comparisons using Wald-type contrasts derived from the fixed-effect estimates and their covariance matrix. Two-tailed p-values were computed from the resulting z-statistics, and the Benjamini–Hochberg false discovery rate (FDR) procedure was applied to correct for multiple pairwise comparisons. We also added the original Benjamini and Hochberg reference to the reference list.

In addition, we revised the Statistical Analysis section to explicitly acknowledge that 5-point Likert ratings are ordinal but were treated as approximately continuous variables in the LMM as a pragmatic modeling approximation. We clarified that this approach allowed us to model the repeated-measures structure of the data while accounting for evaluator- and scenario-level variation.

**Changes made:**

- Statistical Analysis section: clarified the LMM specification, Likert-scale treatment, and post-hoc pairwise comparison procedure.
- Results section: revised the post-hoc reporting to consistently describe Wald-type contrasts with Benjamini–Hochberg FDR correction.

**Comment 2:** *There remains a small but important wording inconsistency in the evaluation procedure. The manuscript states that participants were shown “a single, anonymized LLM-generated response (blind single-evaluation),” but it also states that each session involved evaluating the outputs of all five models across the five questionnaire items. I understand the likely intended meaning, but the unit of evaluation and the session structure should be described more clearly to avoid confusion.*

**Response:** We thank the reviewer for identifying this important wording issue. We have revised the Evaluation Procedure section to clearly distinguish between the unit of evaluation and the session structure.

In the revised manuscript, we explain that each trial presented participants with one workplace scenario prompt and one anonymized LLM-generated response. The model identity was hidden to prevent brand bias. Within each session, participants sequentially completed multiple independent trials, in which the outputs of all five models were evaluated one at a time using the five questionnaire items (Q1–Q5). This revision clarifies that “single-evaluation” referred to the presentation of one response per trial, not to a session containing only one response.

**Changes made:**

- Evaluation Procedure section: revised the description of the trial-level presentation and session-level structure.

**Comment 3:** *The data availability statement is substantially improved and appears to meet the journal’s policy, since the authors provide a public GitHub repository containing prompts, scripts, cleaned human evaluation data, and statistical analysis files. However, for long-term reproducibility, I encourage the authors to provide a versioned archival record (for example, a DOI-backed repository snapshot) or at least specify the repository version/commit hash in the final manuscript.*

**Response:** We thank the reviewer for this helpful suggestion. We have updated the Data Availability Statement in the submission system to include version-specific reproducibility information. Specifically, the statement now provides a DOI-backed Zenodo archive containing the prompts, scripts, cleaned human evaluation data, and statistical analysis files underlying the findings reported in the manuscript.

This revision ensures that readers can identify and access the exact version of the materials underlying the findings reported in the manuscript.

**Changes made:**

- Data Availability Statement in the submission system: added the Zenodo DOI for the version used in this revision.

**Comment 4:** *Finally, the manuscript is generally intelligible and written in acceptable academic English, but it still contains minor language issues that should be corrected before publication. For example, in the Discussion, “these competitive holistic scores is driven” should be revised to “are driven.” A final language-polishing pass would improve readability.*

**Response:** We thank the reviewer for pointing out the remaining language issues. We corrected the specific grammatical error in the Discussion section (“these competitive holistic scores is driven” → “these competitive holistic scores are driven”) and conducted a final language-polishing pass throughout the manuscript. In particular, we revised several sentences to improve subject–verb agreement, sentence structure, clarity, and consistency. We also softened several overly strong expressions to ensure that the conclusions remain appropriately supported by the data.

**Changes made:**

- Discussion section: corrected the subject–verb agreement issue identified by the reviewer.
- Introduction, Results, Discussion, and Conclusion sections: revised wording for clarity, precision, and readability.

**Response to Reviewer #4**

**Comment:** *The paper thoroughly and appropriately addressed all of the revision points that had been raised during the earlier review process, taking each comment and suggestion into careful consideration and implementing the necessary changes in a complete and satisfactory manner.*

**Response:** We sincerely thank the reviewer for the positive evaluation and for confirming that the previous revision points were addressed satisfactorily. We greatly appreciate the reviewer’s careful reading and constructive feedback.

Closing statement

We again thank the Academic Editor and all reviewers for their constructive comments. We believe that the revised manuscript is now clearer, more precise in its statistical reporting, and more reproducible. We hope that the revised version is suitable for publication in PLOS ONE.

Sincerely,

Eiji Aramaki, PhD

Nara Institute of Science and Technology

8916-5 Takayama-cho, Ikoma, Nara 630-0192, JAPAN

+81-743-72-5250

[aramaki@is.naist.jp](mailto:aramaki@is.naist.jp) (On behalf of all authors)
