# Supplementary material for: Evaluating the cultural alignment of multilingual LLMs in typical Japanese workplace scenarios
Source: PLoS One. 2026 Jul 27;21(7):e0338524. doi: 10.1371/journal.pone.0338524 (PMC13405109; doi:10.1371/journal.pone.0338524)
Supplement: Peer review history

# Peer review: Decision Letter 0

**DOI:** 10.1371/journal.pone.0338524.r001

**Authors:** Mohammad Salah Hassan

28 Jan 2026

PONE-D-25-61951

From text to culture: assessing Japanese workplace cultural adaptability in multilingual large language models

PLOS One

Dear Dr. Aramaki,

Thank you for submitting your manuscript to PLOS ONE. After careful consideration, we feel that it has merit but does not fully meet PLOS ONE’s publication criteria as it currently stands. Therefore, we invite you to submit a revised version of the manuscript that addresses the points raised during the review process.

Please submit your revised manuscript by Mar 14 2026 11:59PM. If you will need more time than this to complete your revisions, please reply to this message or contact the journal office at plosone@plos.org. When you're ready to submit your revision, log on to https://www.editorialmanager.com/pone/ and select the 'Submissions Needing Revision' folder to locate your manuscript file.

Please include the following items when submitting your revised manuscript:

- A letter that responds to each point raised by the academic editor and reviewer(s). You should upload this letter as a separate file labeled 'Response to Reviewers'.
- A marked-up copy of your manuscript that highlights changes made to the original version. You should upload this as a separate file labeled 'Revised Manuscript with Track Changes'.
- An unmarked version of your revised paper without tracked changes. You should upload this as a separate file labeled 'Manuscript'.

If you would like to make changes to your financial disclosure, please include your updated statement in your cover letter. Guidelines for resubmitting your figure files are available below the reviewer comments at the end of this letter.

If applicable, we recommend that you deposit your laboratory protocols in protocols.io to enhance the reproducibility of your results. Protocols.io assigns your protocol its own identifier (DOI) so that it can be cited independently in the future. For instructions see: https://journals.plos.org/plosone/s/submission-guidelines#loc-laboratory-protocols. Additionally, PLOS ONE offers an option for publishing peer-reviewed Lab Protocol articles, which describe protocols hosted on protocols.io. Read more information on sharing protocols at https://plos.org/protocols?utm_medium=editorial-email&utm_source=authorletters&utm_campaign=protocols.

We look forward to receiving your revised manuscript.

Kind regards,

Mohammad Salah Hassan, Ph.D

Academic Editor

PLOS One

Journal Requirements:

When submitting your revision, we need you to address these additional requirements.

1. Please ensure that your manuscript meets PLOS ONE's style requirements, including those for file naming. The PLOS ONE style templates can be found at

https://journals.plos.org/plosone/s/file?id=wjVg/PLOSOne_formatting_sample_main_body.pdf and

https://journals.plos.org/plosone/s/file?id=ba62/PLOSOne_formatting_sample_title_authors_affiliations.pdf.

2. Please note that PLOS One has specific guidelines on code sharing for submissions in which author-generated code underpins the findings in the manuscript. In these cases, we expect all author-generated code to be made available without restrictions upon publication of the work. Please review our guidelines at https://journals.plos.org/plosone/s/materials-and-software-sharing#loc-sharing-code and ensure that your code is shared in a way that follows best practice and facilitates reproducibility and reuse.

3. Thank you for stating in your Funding Statement:

“This work was supported by the Cross-ministerial Strategic Innovation Promotion Program (SIP) on 'Integrated Health Care System' (Grant Number JPJ012425) awarded to E.A.. The funders had no role in study design, data collection and analysis, decision to publish, or preparation of the manuscript.”

Please provide an amended statement that declares *all* the funding or sources of support (whether external or internal to your organization) received during this study, as detailed online in our guide for authors at http://journals.plos.org/plosone/s/submit-now.  Please also include the statement “There was no additional external funding received for this study.” in your updated Funding Statement.

Please include your amended Funding Statement within your cover letter. We will change the online submission form on your behalf.

4. We note that you have indicated that there are restrictions to data sharing for this study. For studies involving human research participant data or other sensitive data, we encourage authors to share de-identified or anonymized data. However, when data cannot be publicly shared for ethical reasons, we allow authors to make their data sets available upon request. For information on unacceptable data access restrictions, please see http://journals.plos.org/plosone/s/data-availability#loc-unacceptable-data-access-restrictions.

Before we proceed with your manuscript, please address the following prompts:

a) If there are ethical or legal restrictions on sharing a de-identified data set, please explain them in detail (e.g., data contain potentially identifying or sensitive patient information, data are owned by a third-party organization, etc.) and who has imposed them (e.g., a Research Ethics Committee or Institutional Review Board, etc.). Please also provide contact information for a data access committee, ethics committee, or other institutional body to which data requests may be sent.

b) If there are no restrictions, please upload the minimal anonymized data set necessary to replicate your study findings to a stable, public repository and provide us with the relevant URLs, DOIs, or accession numbers. Please see http://www.bmj.com/content/340/bmj.c181.long for guidelines on how to de-identify and prepare clinical data for publication. For a list of recommended repositories, please see https://journals.plos.org/plosone/s/recommended-repositories. You also have the option of uploading the data as Supporting Information files, but we would recommend depositing data directly to a data repository if possible.

Please update your Data Availability statement in the submission form accordingly.

5. In the online submission form you indicate that your data is not available for proprietary reasons and have provided a contact point for accessing this data. Please note that your current contact point is a co-author on this manuscript. According to our Data Policy, the contact point must not be an author on the manuscript and must be an institutional contact, ideally not an individual. Please revise your data statement to a non-author institutional point of contact, such as a data access or ethics committee, and send this to us via return email. Please also include contact information for the third party organization, and please include the full citation of where the data can be found.

6. Thank you for stating the following in the Competing Interests/Financial Disclosure section:

“This work was supported by the Cross-ministerial Strategic Innovation Promotion Program (SIP) on 'Integrated Health Care System' (Grant Number JPJ012425) awarded to E.A.. The funders had no role in study design, data collection and analysis, decision to publish, or preparation of the manuscript.”

We note that one or more of the authors are employed by a commercial company: name of commercial company.

a.        Please provide an amended Funding Statement declaring this commercial affiliation, as well as a statement regarding the Role of Funders in your study. If the funding organization did not play a role in the study design, data collection and analysis, decision to publish, or preparation of the manuscript and only provided financial support in the form of authors' salaries and/or research materials, please review your statements relating to the author contributions, and ensure you have specifically and accurately indicated the role(s) that these authors had in your study. You can update author roles in the Author Contributions section of the online submission form.

Please also include the following statement within your amended Funding Statement.

“The funder provided support in the form of salaries for authors [insert relevant initials], but did not have any additional role in the study design, data collection and analysis, decision to publish, or preparation of the manuscript. The specific roles of these authors are articulated in the ‘author contributions’ section.”

If your commercial affiliation did play a role in your study, please state and explain this role within your updated Funding Statement.

b.  Please also provide an updated Competing Interests Statement declaring this commercial affiliation along with any other relevant declarations relating to employment, consultancy, patents, products in development, or marketed products, etc.

Within your Competing Interests Statement, please confirm that this commercial affiliation does not alter your adherence to all PLOS ONE policies on sharing data and materials by including the following statement: "This does not alter our adherence to  PLOS ONE policies on sharing data and materials.” (as detailed online in our guide for authors http://journals.plos.org/plosone/s/competing-interests) . If this adherence statement is not accurate and  there are restrictions on sharing of data and/or materials, please state these. Please note that we cannot proceed with consideration of your article until this information has been declared.

Please include both an updated Funding Statement and Competing Interests Statement in your cover letter. We will change the online submission form on your behalf.

7. If the reviewer comments include a recommendation to cite specific previously published works, please review and evaluate these publications to determine whether they are relevant and should be cited. There is no requirement to cite these works unless the editor has indicated otherwise.

Additional Editor Comments (if provided):

Dear Dr. Aramaki,

Thank you for your patience while we conducted the peer review for your manuscript, "From text to culture: assessing Japanese workplace cultural adaptability in multilingual large language models."

The reviewers and I have now completed our evaluation. We all agree that your focus on how LLMs navigate the specific nuances of Japanese workplace culture is a timely and valuable contribution to the field. However, the reviewers have raised several substantial concerns that need to be addressed before we can move forward with publication.

Based on these reports, I am requesting a Major Revision.

In your revision, please pay particular attention to the following:

Reviewers 1 and 2 have noted significant areas where the methodology needs more technical depth and a clearer explanation of the evaluation metrics used.

Reviewer 3 has provided several smaller, yet important, suggestions regarding the linguistic framing and clarity of your results.

When you submit your revised version, please include a detailed "Response to Reviewers" document. In this file, please list each reviewer's comment followed by your specific update or your rebuttal if you disagree with a point. This helps me and the reviewers track the improvements quickly.

I look forward to seeing how you strengthen the paper.

Best regards,

Mohammad Salah Hassan, Ph.D Academic Editor PLOS ONE

[Note: HTML markup is below. Please do not edit.]

Reviewers' comments:

Reviewer's Responses to Questions

**Comments to the Author**

1. Is the manuscript technically sound, and do the data support the conclusions?

The manuscript must describe a technically sound piece of scientific research with data that supports the conclusions. Experiments must have been conducted rigorously, with appropriate controls, replication, and sample sizes. The conclusions must be drawn appropriately based on the data presented.

Reviewer #1: Yes

Reviewer #2: Partly

Reviewer #3: Yes

**********

2. Has the statistical analysis been performed appropriately and rigorously?

Reviewer #1: Yes

Reviewer #2: Yes

Reviewer #3: Yes

**********

3. Have the authors made all data underlying the findings in their manuscript fully available?

The PLOS Data policy requires authors to make all data underlying the findings described in their manuscript fully available without restriction, with rare exception (please refer to the Data Availability Statement in the manuscript PDF file). The data should be provided as part of the manuscript or its supporting information, or deposited to a public repository. For example, in addition to summary statistics, the data points behind means, medians and variance measures should be available. If there are restrictions on publicly sharing data—e.g. participant privacy or use of data from a third party—those must be specified.

Reviewer #1: Yes

Reviewer #2: No

Reviewer #3: Yes

**********

4. Is the manuscript presented in an intelligible fashion and written in standard English?

PLOS ONE does not copyedit accepted manuscripts, so the language in submitted articles must be clear, correct, and unambiguous. Any typographical or grammatical errors should be corrected at revision, so please note any specific errors here.

Reviewer #1: Yes

Reviewer #2: Yes

Reviewer #3: Yes

**********

5. Review Comments to the Author

Please use the space provided to explain your answers to the questions above. You may also include additional comments for the author, including concerns about dual publication, research ethics, or publication ethics. (Please upload your review as an attachment if it exceeds 20,000 characters)

Reviewer #1: Journal: PLOS ONE

Manuscript ID: PONE-D-25-61951

Title: From text to culture: assessing Japanese workplace cultural adaptability in multilingual large language models

This manuscript addresses a timely and important question: the capacity of multilingual LLMs to adapt not only linguistically but culturally to a specific high-context environment—the Japanese workplace. The study is well-motivated, employs a thoughtfully designed dual-framework evaluation (JWCAS + three-layer diagnostic analysis), and yields intriguing findings, notably that certain multilingual models (GLM, Phi) outperform a native Japanese model (LLM-jp).

Recommendation:

I recommend Major Revision. The work has clear potential to influence both LLM evaluation practices and training strategies for culturally-aware AI. However, several substantial concerns must be addressed before publication, primarily regarding: Theoretical grounding and validation of the cultural framework and layer mapping. Methodological transparency and robustness, particularly in sampling, rater variability, and statistical reporting. Interpretative discipline, as the central explanation (“abstract pragmatic transfer”) remains speculative and lacks direct empirical support. The revisions required are significant but feasible, and if adequately addressed, the paper will be a valuable contribution to the field.

Comments:

1. While the integration of Hofstede’s dimensions with a three-layer communicative competence model is innovative, the mapping of questionnaire items (Q1–Q4) to specific layers (Table 2) appears to be post-hoc and lacks empirical validation. This undermines the diagnostic conclusions about where models succeed or fail.

• Provide a clear, principled justification for each item-layer assignment, preferably grounded in intercultural communication theory.

• Conduct and report a factor analysis (or similar) to empirically test whether the items cluster as proposed, or explicitly acknowledge the mapping as a theory-driven analytical lens rather than a validated construct.

• Briefly acknowledge known critiques of Hofstede’s model (static dimensions, national essentialism) and justify its use here despite those limitations.

2. The paper’s key interpretive claim—that GLM/Phi’s superior performance stems from “abstract pragmatic transfer” due to diverse multilingual training, while LLM-jp’s shortfalls reflect “overfitting to linguistic form”—is intriguing but remains hypothetical. No evidence is presented regarding training data composition, architectural differences, or internal model representations.

• Reframe this explanation as a plausible hypothesis rather than a supported conclusion.

• Discuss alternative explanations (e.g., differences in instruction tuning, reinforcement learning from human feedback with diverse annotators, model architecture, or the possibility that Chinese training data shares certain pragmatic norms with Japanese workplaces).

• If possible, add limited analyses (e.g., probing for cultural concepts, comparing models with similar architecture but different training data) to provide more direct evidence; otherwise, highlight this as a critical direction for future research.

3. The reported standard deviations (0.8–0.9 on a 5-point scale) indicate substantial disagreement among raters, which is noted but not analyzed. Additionally, the participant pool is heavily skewed toward older workers (≈80% aged 40+), which may bias norms toward more traditional interpretations of hierarchy, gender roles, or formality.

• Perform a post-hoc analysis of rater disagreement: break down variability by dimension, model, and prompt to identify patterns.

• Discuss how demographic skew (age, possibly industry/role) might influence ratings and limit generalizability.

• Consider adding a qualitative analysis of rater comments to understand sources of disagreement.

4. The selection of the 1,718 evaluations from a larger pool is insufficiently described. The criteria for “representative sampling” are unclear, and potential imbalances across models/dimensions could affect comparability.

• Clearly detail the sampling procedure: e.g., “We randomly selected X responses per model per dimension, stratifying by prompt.”

• Report the total number of generated responses and the distribution of evaluations across models and dimensions in a supplementary table.

• Discuss any potential confounding factors (e.g., different raters evaluating different models).

5.

• Include 1–2 example prompts and model responses in the main text to illustrate the evaluation task and the kinds of differences observed.

• Figure 2 (dumbbell plot): Replace min–max bars with 95% confidence intervals for clearer statistical interpretation.

• Table 3: Add a column for standard deviations alongside means and CIs.

• Briefly discuss the real-world consequences of cultural misalignment in workplace LLM applications (e.g., erosion of trust, communication breakdowns, perpetuation of stereotypes).

• Define key terms (e.g., “pragmatic failure”) upon first use for interdisciplinary readers.

• Ensure acronyms (PDI, IDV, MAS, etc.) are spelled out in the abstract or early in the introduction.

Reviewer #2: Reviewer Comments to Authors

Manuscript ID: PONE-D-25-61951

Manuscript Title: From Text to Culture: Assessing Japanese Workplace Cultural Adaptability in Multilingual Large Language Models

Journal: PLOS ONE

Overall Recommendation: Major Revision

Dear Authors,

Thank you for submitting your manuscript to PLOS ONE. I have reviewed your study on assessing Japanese workplace cultural adaptability in multilingual large language models. The research addresses a timely and practically significant topic. However, several issues need to be addressed before the manuscript can be considered for publication. Please find my detailed comments below.

Major Comments

1.Research Significance and Rationale

The study addresses a highly relevant and practically valuable topic. Given the unique communication norms in Japanese workplace culture, the research focus is well-targeted. The study fills an important gap in the existing literature, as most LLM evaluations have concentrated on Western cultural contexts, with systematic assessments of Japanese workplace culture remaining relatively scarce.

Suggestion: The manuscript does not sufficiently explain why Japanese workplace culture was selected over other non-Western cultures (e.g., Chinese, Indian). I recommend that the authors strengthen the justification for this specific cultural focus in the Introduction section.

2.Research Design and Execution

The overall research design is reasonable; however, several concerns should be addressed:

(1)Insufficient prompt design details: The manuscript does not provide complete prompt templates, showing only partial examples (Table 1). There is a lack of detailed explanation regarding the prompt design process and the degree of standardization applied.

(2)Single sampling issue: Each scenario was sampled only once (single run), which does not account for the inherent randomness in LLM outputs. Although temperature settings were mentioned as 0 or default values, default values may vary across different models, potentially affecting reproducibility.

(3)Limited number of evaluation scenarios: Only seven virtual scenarios were used, which is a relatively small sample size and may limit the generalizability of the conclusions.

Suggestions:

Conduct multiple sampling runs to verify the stability of results. Provide complete prompt templates along with the rationale for their design.

3.Methodological Novelty

The methodology employed is relatively traditional but appears suitable for the research objectives.

Suggestions: Consider introducing more contemporary evaluation frameworks or validating findings against traditional methods. For example:

(1)Employ the “LLM-as-a-judge” approach as a supplementary evaluation method

(2)Adopt multi-turn dialogue evaluation protocols

(3)Utilize established cultural dimension frameworks (e.g., Hofstede’s dimensions) for systematic mapping of cultural constructs

4. Sampling Methods The sampling approach presents notable limitations:

Scenario Sampling:

(1)The selection of seven scenarios was based on the researchers’ “work experience and expertise,” lacking guidance from a systematic theoretical framework.

(2)The representativeness and coverage of the scenario selection are not explained.

(3)The limited number of scenarios may not comprehensively cover key dimensions of Japanese workplace culture.

Model Sampling:

(1)The selection of 10 mainstream LLMs provides reasonable coverage.

(2)However, some model versions may not be the most current (e.g., GPT-4o and Claude 3.5 Sonnet have received updates since).

Evaluator Sampling:

(1)Only three evaluators were used, which is a relatively small sample.

(2)Evaluator backgrounds are not described in sufficient detail (e.g., years of experience working in Japanese companies, language proficiency levels).

(3)It is unclear whether evaluators received standardized training.

Suggestions:

(1)Increase the number of evaluators or provide more detailed descriptions of evaluator qualifications.

(2)Adopt a more systematic framework for scenario selection.

4.Reliability and Validity

Reliability is acceptable, but validity requires strengthening.

Concerns:

(1)The reliability for cultural appropriateness (0.61) is lower than that for linguistic accuracy, suggesting that the evaluation criteria for this dimension may not be sufficiently clear.

(2)The reliability calculations presented in Table 7 are somewhat confusing; the authors should more clearly distinguish reliability across different dimensions and languages.

(3)Validity relies primarily on expert judgment but lacks systematic validation.

Suggestions:

(1)Provide more detailed evaluation criteria and training materials used for the raters.

(2)Reference or establish a more systematic theoretical framework to support the measurement of cultural appropriateness.

5.Data Analysis

The data analysis is fundamentally sound but could be more rigorous.

Concerns:

(1)Qualitative analysis: The coding process lacks transparency, and inter-coder reliability is not reported.

(2)Missing interaction effects: The interaction effects among model type, language, and scenario merit exploration.

Suggestions:

(1)Consider employing multilevel modeling analysis.

(2)Provide detailed documentation of the qualitative coding process and reliability checks.

7. Discussion The discussion is adequate in breadth but lacks depth.

Concerns:

(1)Unclear theoretical contributions: The manuscript does not clearly articulate the study’s theoretical contributions to the field of cross-cultural AI research.

(2)Insufficient dialogue with existing research: The discussion lacks in-depth comparison with related studies cited in the literature review (e.g., Cao et al., Masoud et al.).

(3)Superficial limitations discussion: While sample size limitations are mentioned, other important limitations are not adequately discussed (e.g., subjectivity of evaluation, ecological validity of scenarios).

(4)Insufficient alternative explanations: The observed cross-linguistic differences lack multi-perspective interpretation.

Suggestions:

(1)Strengthen the dialogue with existing theoretical frameworks.

(2)Deepen the multi-perspective interpretation of research findings.

(3)Expand the discussion of study limitations.

Summary

This manuscript addresses an important and timely research question regarding cultural adaptability of LLMs in Japanese workplace contexts. The multi-model, multi-language comparative design is commendable, and the finding regarding the dissociation between linguistic competence and cultural appropriateness is particularly noteworthy. However, the manuscript requires substantial revisions to address concerns regarding sampling adequacy, measurement reliability (particularly for cultural appropriateness), theoretical grounding, and depth of discussion. I encourage the authors to carefully address these issues in their revision.

Reviewer Decision: Major Revision Required

Reviewer #3: 1. There is suggestion that the authors may revise the title to improve clarity and conciseness. In particular, the authors are encouraged to consider “Japanese Workplace Cultural Adaptability in Multilingual Language Models” or “Cultural Sensitivity of Multilingual LLMs in Japanese Work Contexts” as alternative titles.

2. The abstract does not report essential methodological details, including the use of crowdsourced human evaluators, approximate sample size, and study limitations, which should be added for transparency.

The research gap is not explicitly articulated and should be clearly positioned in relation to existing studies on cultural bias and cultural alignment in LLMs.

3. The manuscript lacks a concise and structured statement of its key contributions, which should be added.

4. Well-documented critiques of Hofstede’s model, including cultural essentialism and contextual rigidity, are not addressed and should be acknowledged.

5. The sampling and evaluation pipeline requires clearer explanation, particularly regarding the transition from generated model outputs to the final set of human-evaluated responses.

6. The relatively small mean differences between models are not sufficiently contextualized in terms of their real-world relevance to workplace communication.

7. Interpretations related to training data diversity, model overfitting, and “abstract pragmatic transfer” are speculative and not empirically tested, and should therefore be framed more cautiously as hypotheses.

8. The manuscript does not address the temporal validity of the findings despite the rapid evolution of LLM architectures.

9. Greater inclusion of peer-reviewed literature from cross-cultural communication, organizational behavior, and AI ethics is required to strengthen the scholarly foundation.

10. The manuscript requires clearer articulation of its contributions, stronger theoretical reflexivity, improved methodological transparency, more cautious interpretation of results, and broader engagement with peer-reviewed literature before it can be considered for publication.

**********

6. PLOS authors have the option to publish the peer review history of their article (what does this mean?). If published, this will include your full peer review and any attached files.

If you choose “no”, your identity will remain anonymous but your review may still be made public.

**Do you want your identity to be public for this peer review?**  For information about this choice, including consent withdrawal, please see our Privacy Policy.

Reviewer #1: **Yes:**  Reza Kazemi

Reviewer #2: No

Reviewer #3: **Yes:**  Ayesha Noreen

**********

[NOTE: If reviewer comments were submitted as an attachment file, they will be attached to this email and accessible via the submission site. Please log into your account, locate the manuscript record, and check for the action link "View Attachments". If this link does not appear, there are no attachment files.]

To ensure your figures meet our technical requirements, please review our figure guidelines: https://journals.plos.org/plosone/s/figures

You may also use PLOS’s free figure tool, NAAS, to help you prepare publication quality figures: https://journals.plos.org/plosone/s/figures#loc-tools-for-figure-preparation.

NAAS will assess whether your figures meet our technical requirements by comparing each figure against our figure specifications.

Attachment Submitted filename: Final Comments.docx

# Author comment: Author response to Decision Letter 1

**DOI:** 10.1371/journal.pone.0338524.r002

14 Mar 2026

Dear Academic Editors of PLOS ONE,

Thank you for giving us the opportunity to submit a revised draft of our manuscript. We deeply appreciate the time and effort that you and the reviewers have dedicated to evaluating our work. The constructive feedback has been instrumental in significantly improving the rigor, clarity, and depth of our paper.

We have carefully considered all comments and revised the manuscript accordingly. Below, we provide a point-by-point response to each reviewer's comments.

________________________________

General Response: Clarification on the Updated Statistical Data

________________________________

Before addressing the specific comments, we would like to transparently report a correction made to the statistical results in this revised manuscript.

During the revision process, we discovered a transcription oversight in our initial submission. The original Japanese Workplace Cultural Alignment Score (JWCAS) values reported in the text were inadvertently pulled from a preliminary calculation that averaged all questions (Q1–Q5), rather than the strictly defined holistic metric (Q5 only) as stated in our methodology.

To rectify this, we have corrected the JWCAS calculations to exclusively reflect Q5. Consequently, we have also updated the corresponding statistical analysis throughout the Results section, employing a rigorous Linear Mixed-Effects Model (LMM) with FDR correction to ensure total accuracy and methodological alignment.

Importantly, while the specific numerical values have changed, this robust re-analysis has strengthened our core findings. The updated results explicitly confirm that the leading multilingual models (Phi and GLM) achieved overall cultural alignment scores that rivaled or even significantly surpassed the native Japanese model (LLM-jp). This provides even stronger empirical support for our hypotheses regarding the "Illusion of Fluency" and "Abstract Pragmatic Transfer" discussed in the paper.

To ensure complete transparency and to support the PLOS ONE data policy, we have open-sourced our entire analytical pipeline. All cleaned human evaluation data and code containing our statistical calculations are now publicly available in our repository

________________________________

Point-by-Point Responses to Reviewers

________________________________

Reviewer #1

Comment 1.1: While the integration of Hofstede’s dimensions with a three-layer communicative competence model is innovative, the mapping of questionnaire items (Q1–Q4) to specific layers (Table 2) appears to be post-hoc and lacks empirical validation... Provide a clear, principled justification... or explicitly acknowledge the mapping as a theory-driven analytical lens. Briefly acknowledge known critiques of Hofstede’s model.

Response: We thank the reviewer for this excellent point. We completely agree that the mapping is explanatory rather than psychometrically validated. As suggested, we have explicitly reframed this mapping as a "theory-driven analytical lens" used to dissect qualitative differences in model performance, rather than an absolute psychometric construct. Furthermore, we have added explicit acknowledgments of the critiques of Hofstede’s model (e.g., cultural essentialism, static monoliths) in both the Introduction and Limitations sections.

--------------------------------

Comment 1.2: The paper’s key interpretive claim—that GLM/Phi’s superior performance stems from “abstract pragmatic transfer”... remains hypothetical. No evidence is presented regarding training data composition... Reframe this explanation as a plausible hypothesis.

Response: We appreciate this vital methodological critique. We have revised the manuscript to strictly frame "abstract pragmatic transfer" as a hypothesis rather than a proven conclusion. In the Discussion section, we have also added a paragraph discussing alternative factors that could drive these performance gaps, such as differences in Reinforcement Learning from Human Feedback (RLHF) datasets and specific instruction-tuning strategies.

--------------------------------

Comment 1.3: The reported standard deviations (0.8–0.9 on a 5-point scale) indicate substantial disagreement among raters... Perform a post-hoc analysis of rater disagreement... Discuss how demographic skew (age, possibly industry/role) might influence ratings.

Response: We thank the reviewer for this perceptive comment. To address the request for a breakdown of rater variability, we point to our newly added S3 Table to the Supporting Information. This table details the Standard Deviations (SDs) across all specific models and diagnostic dimensions. The analysis reveals that high variability (SDs generally between 0.8 and 1.0) is a consistent feature across almost all models and cultural dimensions, indicating that this disagreement is likely an inherent characteristic of evaluating subjective cultural norms rather than an anomaly specific to one model or prompt. Furthermore, we have explicitly discussed this high variability, along with the demographic skew of our crowdsourced sample (which leans toward experienced, older professionals), in the Limitations section, noting how it might bias interpretations toward more traditional workplace norms.

--------------------------------

Comment 1.4: The selection of the 1,718 evaluations from a larger pool is insufficiently described. The criteria for “representative sampling” are unclear... Clearly detail the sampling procedure.

Response: We apologize for the lack of clarity. We have expanded the Methods section to explicitly detail the stratified random sampling procedure. We clarified that a total of 30,000 texts were generated (1,000 per model per dimension), from which responses were dynamically and randomly sampled for human evaluators, yielding the final 1,718 valid sessions. Furthermore, exactly as requested, we have added S4 Table to the Supporting Information, which clearly reports the distribution of these 1,718 evaluations across all models and Hofstede dimensions.

--------------------------------

Comment 1.5: Include 1–2 example prompts and model responses in the main text... Replace min–max bars with 95% confidence intervals... Table 3: Add a column for standard deviations... Briefly discuss the real-world consequences... Define key terms... Ensure acronyms are spelled out.

Response: We sincerely thank the reviewer for these excellent suggestions, which have greatly enhanced the readability and precision of our manuscript. We have addressed all of these points as follows:

1. Example Prompts: We have integrated a detailed descriptive example of a PDI scenario (correcting a superior's mistake) directly into the Methods section (Page 4, Lines 105-113) to illustrate the divergence between grammatical politeness and pragmatic strategy.

2. Table 3 (SDs): We have added the Standard Deviations (SDs) alongside the means and CIs for the holistic scores (Q5) in Table 3, as requested.

3. Figure 2 (CIs): Figure 2 visualizes the diagnostic sub-scores (Q1-Q4). We encountered a visualization challenge here: plotting five overlapping 95% CIs on a single horizontal axis for every sub-score category rendered the dumbbell plot highly cluttered and unreadable. To ensure visual clarity while strictly adhering to your request for statistical rigor, we retained the current plot format to illustrate macro-trends. However, we have calculated the precise Means, SDs, and 95% CIs for all diagnostic sub-scores (Q1-Q4) and compiled them into a new supplementary table (S3 Table). We have added a note in the Figure 2 caption directing readers to this table for the complete statistical intervals.

4. Real-world Consequences: We have expanded the Implications subsection to explicitly discuss how pragmatic failures (despite flawless grammar) can cause critical interpersonal friction in high-context environments.

5. Definitions & Acronyms: We have defined "Pragmatic Failure" upon its first use in both the Abstract and the Discussion, and we have ensured all Hofstede dimension acronyms are spelled out upon their initial introduction.

________________________________

Reviewer #2

(Note to authors: Several points raised by Reviewer 2 appear to reference experimental parameters that differ significantly from our actual methodology. We respectfully clarify our actual study design below.)

--------------------------------

Comment 2.1: The manuscript does not sufficiently explain why Japanese workplace culture was selected over other non-Western cultures (e.g., Chinese, Indian). I recommend that the authors strengthen the justification for this specific cultural focus in the Introduction section.

Response: We thank the reviewer for this insightful recommendation. We have expanded the Introduction section to explicitly justify our focus on the Japanese workplace. We clarified that Japan serves as a uniquely rigorous "stress-test" environment for LLMs. Unlike many other non-Western cultures, Japanese workplace communication features a highly codified, rigid socio-linguistic layer (Honorifics / Keigo) that frequently diverges from the underlying pragmatic intent (the high-context necessity of "reading the air" or Kuuki wo yomu). This unique dichotomy makes it the perfect cultural environment to test our core hypothesis: distinguishing between an LLM's surface-level "Illusion of Fluency" and its true, deep socio-pragmatic alignment.

--------------------------------

Comment 2.2: Insufficient prompt design details... Single sampling issue: Each scenario was sampled only once (single run)... Limited number of evaluation scenarios: Only seven virtual scenarios were used.

Response: We respectfully wish to clarify the scale of our study design, which may have been misunderstood in our initial description. Our study did not use a single run or only seven scenarios. As now detailed explicitly in our revised Methods section, we constructed 30 distinct evaluation scenarios (5 unique scenarios for each of the 6 Hofstede dimensions). Furthermore, each model generated 1,000 responses per dimension, resulting in a total corpus of 30,000 generated responses, mitigating the randomness of single-run outputs. To address the request for prompt details, we have also added a descriptive example of a PDI scenario directly into the main text, and the complete English translations of all 30 prompt templates are provided in S1 Table.

--------------------------------

Comment 2.3: Evaluator Sampling: Only three evaluators were used, which is a relatively small sample... The reliability for cultural appropriateness (0.61) is lower...

Response: We believe there may be a critical misunderstanding regarding our evaluator pool. Our study utilized a large-scale crowdsourcing approach, yielding 1,718 valid human evaluation sessions, not three evaluators. Because each session evaluated the outputs of all five models across the five questionnaire items, this generated a massive dataset of 42,950 individual Likert-scale rating data points. To make this completely transparent, we have added S4 Table, which maps the distribution of these 1,718 sessions across the cultural dimensions. Additionally, our calculated Intraclass Correlation Coefficient (ICC) for the evaluations is 0.837 (indicating excellent reliability), not 0.61. We have ensured these methodological details are maximally prominent in the revised Methods section.

--------------------------------

Comment 2.4: Consider introducing more contemporary evaluation frameworks... Employ the “LLM-as-a-judge” approach as a supplementary evaluation method. Adopt multi-turn dialogue evaluation protocols.

Response: We deeply appreciate these forward-looking methodological suggestions. While "LLM-as-a-judge" is highly efficient for general instruction-following benchmarks, we deliberately avoided it for this specific study. Recent literature highlights that state-of-the-art LLMs exhibit strong Western cultural biases. Using a potentially culturally biased LLM to evaluate the nuanced, high-context socio-pragmatic behaviors of other models risks circular validation and could mask subtle cultural misalignments. Therefore, we maintained our crowdsourced human evaluation, as native human judgment remains the absolute gold standard for assessing authentic cultural resonance. Regarding multi-turn dialogue, we completely agree it represents the next frontier for cultural evaluation, and we have explicitly added this to our Limitations and Future Work section.

--------------------------------

Comment 2.5: Discussion lacks depth... Unclear theoretical contributions... Superficial limitations discussion.

Response: We thank the reviewer for pushing us to deepen our scholarly discussion. In the revised manuscript, we have significantly expanded the Discussion and Implications sections. We clearly articulate our theoretical contribution: moving beyond single-metric evaluations to a multi-layered diagnostic approach (differentiating Form, Values, and Action). We also expanded the Limitations section to thoroughly discuss the subjectivity and demographic skew of human evaluations, the temporality of LLM architectures, and the critiques of Hofstede's model (e.g., cultural essentialism).

________________________________

Reviewer #3

Comment 3.1: There is a suggestion that the authors may revise the title to improve clarity and conciseness. In particular, the authors are encouraged to consider “Japanese Workplace Cultural Adaptability in Multilingual Language Models” or “Cultural Sensitivity of Multilingual LLMs in Japanese Work Contexts” as alternative titles.

Response: We sincerely thank the reviewer for these excellent suggestions. We entirely agree that a more concise title strengthens the paper. We have revised the title to: "Evaluating the Cultural Alignment of Multilingual LLMs in Typical Japanese Workplace Scenarios", which captures the essence of both of your suggestions while maintaining precision.

--------------------------------

Comments 3.2, 3.3 & 3.10: The abstract does not report essential methodological details... The research gap is not explicitly articulated... The manuscript lacks a concise and structured statement of its key contributions.

Response: We deeply appreciate this structural feedback. We have extensively overhauled the Abstract and the final paragraphs of the Introduction to explicitly address these omissions:

1. Research Gap: We now explicitly state the gap: while existing evaluations predominantly focus on Western contexts or rely on static multiple-choice benchmarks, our study addresses the critical need to evaluate generative, high-context non-Western socio-pragmatic alignment.

2. Methodological Details: We have incorporated the essential figures into the Abstract, specifying the use of crowdsourced human evaluators, the sample size (1,718 valid sessions generating 42,950 rating data points), and the dual-evaluation framework.

3. Key Contributions: We have added a concise statement in both the Abstract and Introduction highlighting our main contribution: demonstrating that holistic evaluation metrics can obscure deep pragmatic deficits (the "Illusion of Fluency"), and that our layer-by-layer diagnosis reveals the distinct strategies models use to achieve alignment.

--------------------------------

Comments 3.4 & 3.9: Well-documented critiques of Hofstede’s model (cultural essentialism) are not addressed... Greater inclusion of peer-reviewed literature from cross-cultural communication is required.

Response: This is a crucial theoretical point. We have explicitly acknowledged the limitations of Hofstede's framework (specifically cultural essentialism and the risk of treating national cultures as static monoliths) in the Introduction and Limitations sections. Furthermore, to strengthen our theoretical reflexivity, we have integrated foundational cross-cultural and pragmatic literature (e.g., Hall, 1976; Meyer, 2014; Ide, 1989; Thomas, 1983). These additions help justify our selection of the Japanese workplace not merely as an application case, but as a uniquely rigorous "stress-test" environment due to its extreme high-context nature and rigid honorific system.

--------------------------------

Attachment Submitted filename: response_letter.docx

# Peer review: Decision Letter 1

**DOI:** 10.1371/journal.pone.0338524.r003

**Authors:** Mohammad Salah Hassan

4 May 2026

PONE-D-25-61951R1

Evaluating the cultural alignment of multilingual LLMs in typical Japanese workplace scenarios

PLOS One

Dear Dr. Aramaki,

Thank you for submitting your manuscript to PLOS ONE. After careful consideration, we feel that it has merit but does not fully meet PLOS ONE’s publication criteria as it currently stands. Therefore, we invite you to submit a revised version of the manuscript that addresses the points raised during the review process.

Please submit your revised manuscript by Jun 18 2026 11:59PM. If you will need more time than this to complete your revisions, please reply to this message or contact the journal office at plosone@plos.org. When you're ready to submit your revision, log on to https://www.editorialmanager.com/pone/ and select the 'Submissions Needing Revision' folder to locate your manuscript file.

Please include the following items when submitting your revised manuscript:

- A letter that responds to each point raised by the academic editor and reviewer(s). You should upload this letter as a separate file labeled 'Response to Reviewers'.
- A marked-up copy of your manuscript that highlights changes made to the original version. You should upload this as a separate file labeled 'Revised Manuscript with Track Changes'.
- An unmarked version of your revised paper without tracked changes. You should upload this as a separate file labeled 'Manuscript'.

If you would like to make changes to your financial disclosure, please include your updated statement in your cover letter. Guidelines for resubmitting your figure files are available below the reviewer comments at the end of this letter.

If applicable, we recommend that you deposit your laboratory protocols in protocols.io to enhance the reproducibility of your results. Protocols.io assigns your protocol its own identifier (DOI) so that it can be cited independently in the future. For instructions see: https://journals.plos.org/plosone/s/submission-guidelines#loc-laboratory-protocols. Additionally, PLOS ONE offers an option for publishing peer-reviewed Lab Protocol articles, which describe protocols hosted on protocols.io. Read more information on sharing protocols at https://plos.org/protocols?utm_medium=editorial-email&utm_source=authorletters&utm_campaign=protocols.

As the corresponding author, your ORCID iD is verified in the submission system and will appear in the published article. PLOS supports the use of ORCID, and we encourage all coauthors to register for an ORCID iD and use it as well. Please encourage your coauthors to verify their ORCID iD within the submission system before final acceptance, as unverified ORCID iDs will not appear in the published article. *Only*  the individual author can complete the verification step; PLOS staff *cannot*  verify ORCID iDs on behalf of authors.

We look forward to receiving your revised manuscript.

Kind regards,

Mohammad Salah Hassan, Ph.D

Academic Editor

PLOS One

**Journal Requirements:**

1. If the reviewer comments include a recommendation to cite specific previously published works, please review and evaluate these publications to determine whether they are relevant and should be cited. There is no requirement to cite these works unless the editor has indicated otherwise.

2. Please review your reference list to ensure that it is complete and correct. If you have cited papers that have been retracted, please include the rationale for doing so in the manuscript text, or remove these references and replace them with relevant current references. Any changes to the reference list should be mentioned in the rebuttal letter that accompanies your revised manuscript. If you need to cite a retracted article, indicate the article’s retracted status in the References list and also include a citation and full reference for the retraction notice.

**Additional Editor Comments:**

Dear Authors,

Thank you for submitting the revised manuscript. The reviewer is satisfied with the substantial improvements and considers the manuscript technically sound. However, before a final decision can be made, please address the remaining minor comments raised by the reviewer carefully.

In particular, please clarify the statistical reporting, resolve the wording inconsistency in the evaluation procedure, strengthen the reproducibility details in the data availability statement, and complete a final language-polishing pass.

Please submit a revised version together with a brief point-by-point response to the reviewer’s remaining comments.

Kind regards,

[Note: HTML markup is below. Please do not edit.]

Reviewers' comments:

Reviewer's Responses to Questions

**Comments to the Author**

1. If the authors have adequately addressed your comments raised in a previous round of review and you feel that this manuscript is now acceptable for publication, you may indicate that here to bypass the “Comments to the Author” section, enter your conflict of interest statement in the “Confidential to Editor” section, and submit your "Accept" recommendation.

Reviewer #1: All comments have been addressed

Reviewer #2: All comments have been addressed

Reviewer #4: All comments have been addressed

**********

2. Is the manuscript technically sound, and do the data support the conclusions?

The manuscript must describe a technically sound piece of scientific research with data that supports the conclusions. Experiments must have been conducted rigorously, with appropriate controls, replication, and sample sizes. The conclusions must be drawn appropriately based on the data presented.

Reviewer #1: Yes

Reviewer #2: Yes

Reviewer #4: Yes

**********

3. Has the statistical analysis been performed appropriately and rigorously?

Reviewer #1: Yes

Reviewer #2: Yes

Reviewer #4: Yes

**********

4. Have the authors made all data underlying the findings in their manuscript fully available?

The PLOS Data policy requires authors to make all data underlying the findings described in their manuscript fully available without restriction, with rare exception (please refer to the Data Availability Statement in the manuscript PDF file). The data should be provided as part of the manuscript or its supporting information, or deposited to a public repository. For example, in addition to summary statistics, the data points behind means, medians and variance measures should be available. If there are restrictions on publicly sharing data—e.g. participant privacy or use of data from a third party—those must be specified.

Reviewer #1: Yes

Reviewer #2: Yes

Reviewer #4: Yes

**********

5. Is the manuscript presented in an intelligible fashion and written in standard English?

PLOS ONE does not copyedit accepted manuscripts, so the language in submitted articles must be clear, correct, and unambiguous. Any typographical or grammatical errors should be corrected at revision, so please note any specific errors here.

Reviewer #1: Yes

Reviewer #2: Yes

Reviewer #4: Yes

**********

6. Review Comments to the Author

Please use the space provided to explain your answers to the questions above. You may also include additional comments for the author, including concerns about dual publication, research ethics, or publication ethics. (Please upload your review as an attachment if it exceeds 20,000 characters)

**Reviewer #1:**  Thank you for your careful and thorough responses to my comments. The revisions are appreciated and have strengthened the paper. I have no further questions.

**Reviewer #2:**  Thank you for the substantial revision. The manuscript is considerably improved, and the authors have addressed most of the major concerns raised in the previous round. In particular, I appreciate the clarification that the Japanese Workplace Cultural Alignment Score (JWCAS) is now correctly based on Q5 only, the expanded explanation of the sampling pipeline (30 scenarios, 30,000 generated responses, 1,718 valid evaluation sessions), the explicit reframing of the three-layer mapping as a theory-driven analytical lens rather than a psychometrically validated construct, and the more cautious treatment of “abstract pragmatic transfer” as a hypothesis rather than an empirically established mechanism. The expanded limitations and data availability statement also strengthen the manuscript.

In my view, the manuscript is now technically much stronger, and the main conclusions are broadly supported at the level of comparative performance patterns. However, I recommend one final round of minor revision before acceptance, mainly for clarity and reporting precision.

First, the statistical reporting should be made more explicit. The manuscript states that post-hoc comparisons were conducted using Tukey’s HSD, but it also states that Benjamini-Hochberg FDR correction was applied. Please clarify exactly how these procedures were combined. In addition, because the outcome consists of 5-point Likert ratings analyzed with an LMM, it would be helpful to briefly justify the treatment of these ratings as approximately continuous, or at least acknowledge this modeling choice as a pragmatic approximation.

Second, there remains a small but important wording inconsistency in the evaluation procedure. The manuscript states that participants were shown “a single, anonymized LLM-generated response (blind single-evaluation),” but it also states that each session involved evaluating the outputs of all five models across the five questionnaire items. I understand the likely intended meaning, but the unit of evaluation and the session structure should be described more clearly to avoid confusion.

Third, the data availability statement is substantially improved and appears to meet the journal’s policy, since the authors provide a public GitHub repository containing prompts, scripts, cleaned human evaluation data, and statistical analysis files. However, for long-term reproducibility, I encourage the authors to provide a versioned archival record (for example, a DOI-backed repository snapshot) or at least specify the repository version/commit hash in the final manuscript.

Finally, the manuscript is generally intelligible and written in acceptable academic English, but it still contains minor language issues that should be corrected before publication. For example, in the Discussion, “these competitive holistic scores is driven” should be revised to “are driven.” A final language-polishing pass would improve readability.

Overall, this is now a much clearer and more rigorous manuscript. My remaining concerns are minor and primarily editorial/reporting in nature. I would be supportive of publication after these final revisions are addressed.

**Reviewer #4:**  The paper thoroughly and appropriately addressed all of the revision points that had been raised during the earlier review process, taking each comment and suggestion into careful consideration and implementing the necessary changes in a complete and satisfactory manner.

**********

7. PLOS authors have the option to publish the peer review history of their article (what does this mean?). If published, this will include your full peer review and any attached files.

If you choose “no”, your identity will remain anonymous but your review may still be made public.

**Do you want your identity to be public for this peer review?**  For information about this choice, including consent withdrawal, please see our Privacy Policy.

Reviewer #1: **Yes:**  Reza Kazemi

Reviewer #2: No

Reviewer #4: No

**********

[NOTE: If reviewer comments were submitted as an attachment file, they will be attached to this email and accessible via the submission site. Please log into your account, locate the manuscript record, and check for the action link "View Attachments". If this link does not appear, there are no attachment files.]

To ensure your figures meet our technical requirements, please review our figure guidelines: https://journals.plos.org/plosone/s/figures

You may also use PLOS’s free figure tool, NAAS, to help you prepare publication quality figures: https://journals.plos.org/plosone/s/figures#loc-tools-for-figure-preparation.

NAAS will assess whether your figures meet our technical requirements by comparing each figure against our figure specifications.

Attachment Submitted filename: Reviewer Comments on Revised Manuscript.docx

# Author comment: Author response to Decision Letter 2

**DOI:** 10.1371/journal.pone.0338524.r004

3 Jun 2026

Dear Academic Editor and Reviewers,

We sincerely thank the Academic Editor and the reviewers for their careful evaluation of our revised manuscript. We are grateful that the reviewers found the manuscript substantially improved and technically sound. In this revision, we have addressed the remaining minor comments by clarifying the statistical reporting, resolving the wording inconsistency in the evaluation procedure, strengthening the reproducibility information in the Data Availability Statement, and conducting a final language-polishing pass.

Below, we provide a point-by-point response to the remaining comments.

________________________________

Response to the Academic Editor

Comment:

The reviewer is satisfied with the substantial improvements and considers the manuscript technically sound. However, before a final decision can be made, please address the remaining minor comments raised by the reviewer carefully. In particular, please clarify the statistical reporting, resolve the wording inconsistency in the evaluation procedure, strengthen the reproducibility details in the data availability statement, and complete a final language-polishing pass.

Response:

We thank the Academic Editor for the positive assessment and for summarizing the remaining issues. In the revised manuscript, we clarified the statistical reporting, corrected the description of the evaluation procedure, updated the Data Availability Statement in the submission system with version-specific reproducibility information, and conducted a final language-polishing pass. We have also revised the manuscript text and reference list where needed.

________________________________

Response to Reviewer #1

Comment:

All comments have been addressed. Thank you for your careful and thorough responses to my comments. The revisions are appreciated and have strengthened the paper. I have no further questions.

Response:

We sincerely thank the reviewer for the positive evaluation and for the helpful comments in the previous round. We are grateful that the reviewer found the revisions satisfactory.

________________________________

Response to Reviewer #2

Comment 1:

The statistical reporting should be made more explicit. The manuscript states that post-hoc comparisons were conducted using Tukey’s HSD, but it also states that Benjamini-Hochberg FDR correction was applied. Please clarify exactly how these procedures were combined. In addition, because the outcome consists of 5-point Likert ratings analyzed with an LMM, it would be helpful to briefly justify the treatment of these ratings as approximately continuous, or at least acknowledge this modeling choice as a pragmatic approximation.

Response:

We thank the reviewer for pointing out this ambiguity. We have clarified the statistical analysis in the revised manuscript. Specifically, the post-hoc analysis did not combine Tukey’s HSD with Benjamini–Hochberg correction. The earlier wording was imprecise, and we have revised it throughout the manuscript.

In the final analysis, after fitting the Linear Mixed-Effects Model (LMM), we conducted pairwise model comparisons using Wald-type contrasts derived from the fixed-effect estimates and their covariance matrix. Two-tailed p-values were computed from the resulting z-statistics, and the Benjamini–Hochberg false discovery rate (FDR) procedure was applied to correct for multiple pairwise comparisons. We also added the original Benjamini and Hochberg reference to the reference list.

In addition, we revised the Statistical Analysis section to explicitly acknowledge that 5-point Likert ratings are ordinal but were treated as approximately continuous variables in the LMM as a pragmatic modeling approximation. We clarified that this approach allowed us to model the repeated-measures structure of the data while accounting for evaluator- and scenario-level variation.

Changes made:

- Statistical Analysis section: clarified the LMM specification, Likert-scale treatment, and post-hoc pairwise comparison procedure.

- Results section: revised the post-hoc reporting to consistently describe Wald-type contrasts with Benjamini–Hochberg FDR correction.

================================

Comment 2:

There remains a small but important wording inconsistency in the evaluation procedure. The manuscript states that participants were shown “a single, anonymized LLM-generated response (blind single-evaluation),” but it also states that each session involved evaluating the outputs of all five models across the five questionnaire items. I understand the likely intended meaning, but the unit of evaluation and the session structure should be described more clearly to avoid confusion.

Response:

We thank the reviewer for identifying this important wording issue. We have revised the Evaluation Procedure section to clearly distinguish between the unit of evaluation and the session structure.

In the revised manuscript, we explain that each trial presented participants with one workplace scenario prompt and one anonymized LLM-generated response. The model identity was hidden to prevent brand bias. Within each session, participants sequentially completed multiple independent trials, in which the outputs of all five models were evaluated one at a time using the five questionnaire items (Q1–Q5). This revision clarifies that “single-evaluation” referred to the presentation of one response per trial, not to a session containing only one response.

Changes made:

- Evaluation Procedure section: revised the description of the trial-level presentation and session-level structure.

================================

Comment 3:

The data availability statement is substantially improved and appears to meet the journal’s policy, since the authors provide a public GitHub repository containing prompts, scripts, cleaned human evaluation data, and statistical analysis files. However, for long-term reproducibility, I encourage the authors to provide a versioned archival record (for example, a DOI-backed repository snapshot) or at least specify the repository version/commit hash in the final manuscript.

Response:

We thank the reviewer for this helpful suggestion. We have updated the Data Availability Statement in the submission system to include version-specific reproducibility information. Specifically, the statement now provides a DOI-backed Zenodo archive containing the prompts, scripts, cleaned human evaluation data, and statistical analysis files underlying the findings reported in the manuscript.

This revision ensures that readers can identify and access the exact version of the materials underlying the findings reported in the manuscript.

Changes made:

- Data Availability Statement in the submission system: added the Zenodo DOI for the version used in this revision.

================================

Comment 4:

Finally, the manuscript is generally intelligible and written in acceptable academic English, but it still contains minor language issues that should be corrected before publication. For example, in the Discussion, “these competitive holistic scores is driven” should be revised to “are driven.” A final language-polishing pass would improve readability.

Response:

We thank the reviewer for pointing out the remaining language issues. We corrected the specific grammatical error in the Discussion section (“these competitive holistic scores is driven” → “these competitive holistic scores are driven”) and conducted a final language-polishing pass throughout the manuscript. In particular, we revised several sentences to improve subject–verb agreement, sentence structure, clarity, and consistency. We also softened several overly strong expressions to ensure that the conclusions remain appropriately supported by the data.

Changes made:

- Discussion section: corrected the subject–verb agreement issue identified by the reviewer.

- Introduction, Results, Discussion, and Conclusion sections: revised wording for clarity, precision, and readability.

________________________________

Response to Reviewer #4

Comment:

The paper thoroughly and appropriately addressed all of the revision points that had been raised during the earlier review process, taking each comment and suggestion into careful consideration and implementing the necessary changes in a complete and satisfactory manner.

Response:

We sincerely thank the reviewer for the positive evaluation and for confirming that the previous revision points were addressed satisfactorily. We greatly appreciate the reviewer’s careful reading and constructive feedback.

________________________________

Closing statement

We again thank the Academic Editor and all reviewers for their constructive comments. We believe that the revised manuscript is now clearer, more precise in its statistical reporting, and more reproducible. We hope that the revised version is suitable for publication in PLOS ONE.

Sincerely,

Eiji Aramaki, PhD

Nara Institute of Science and Technology

8916-5 Takayama-cho, Ikoma, Nara 630-0192, JAPAN

Attachment Submitted filename: response_letter_2.docx

# Peer review: Decision Letter 2

**DOI:** 10.1371/journal.pone.0338524.r005

**Authors:** Mohammad Salah Hassan

13 Jul 2026

Evaluating the cultural alignment of multilingual LLMs in typical Japanese workplace scenarios

PONE-D-25-61951R2

Dear Dr. Authors,

We’re pleased to inform you that your manuscript has been judged scientifically suitable for publication and will be formally accepted for publication once it meets all outstanding technical requirements.

Within one week, you’ll receive an e-mail detailing the required amendments. When these have been addressed, you’ll receive a formal acceptance letter and your manuscript will be scheduled for publication.

An invoice will be generated when your article is formally accepted. Please note, if your institution has a publishing partnership with PLOS and your article meets the relevant criteria, all or part of your publication costs will be covered. Please make sure your user information is up-to-date by logging into Editorial Manager at Editorial Manager® and clicking the ‘Update My Information' link at the top of the page. For questions related to billing, please contact billing support.

If your institution or institutions have a press office, please notify them about your upcoming paper to help maximize its impact. If they’ll be preparing press materials, please inform our press team as soon as possible -- no later than 48 hours after receiving the formal acceptance. Your manuscript will remain under strict press embargo until 2 pm Eastern Time on the date of publication. For more information, please contact onepress@plos.org.

Kind regards,

Mohammad Salah Hassan, Ph.D

Academic Editor

PLOS One

Additional Editor Comments (optional):

Dear Authors,

The reviewers have accepted the revised manuscript and confirmed that the previous concerns were addressed satisfactorily. The manuscript is now suitable for publication.

Kind regards,

Academic Editor

Reviewers' comments:

Reviewer's Responses to Questions

**Comments to the Author**

1. If the authors have adequately addressed your comments raised in a previous round of review and you feel that this manuscript is now acceptable for publication, you may indicate that here to bypass the “Comments to the Author” section, enter your conflict of interest statement in the “Confidential to Editor” section, and submit your "Accept" recommendation.

Reviewer #2: All comments have been addressed

**********

2. Is the manuscript technically sound, and do the data support the conclusions?

The manuscript must describe a technically sound piece of scientific research with data that supports the conclusions. Experiments must have been conducted rigorously, with appropriate controls, replication, and sample sizes. The conclusions must be drawn appropriately based on the data presented.

Reviewer #2: Yes

**********

3. Has the statistical analysis been performed appropriately and rigorously?

Reviewer #2: Yes

**********

4. Have the authors made all data underlying the findings in their manuscript fully available?

The PLOS Data policy requires authors to make all data underlying the findings described in their manuscript fully available without restriction, with rare exception (please refer to the Data Availability Statement in the manuscript PDF file). The data should be provided as part of the manuscript or its supporting information, or deposited to a public repository. For example, in addition to summary statistics, the data points behind means, medians and variance measures should be available. If there are restrictions on publicly sharing data—e.g. participant privacy or use of data from a third party—those must be specified.

Reviewer #2: Yes

**********

5. Is the manuscript presented in an intelligible fashion and written in standard English?

PLOS ONE does not copyedit accepted manuscripts, so the language in submitted articles must be clear, correct, and unambiguous. Any typographical or grammatical errors should be corrected at revision, so please note any specific errors here.

Reviewer #2: Yes

**********

6. Review Comments to the Author

Please use the space provided to explain your answers to the questions above. You may also include additional comments for the author, including concerns about dual publication, research ethics, or publication ethics. (Please upload your review as an attachment if it exceeds 20,000 characters)

Reviewer #2: The authors have carefully addressed my remaining comments. The revised manuscript now clearly explains the post-hoc procedure using Wald-type contrasts with Benjamini-Hochberg FDR correction, acknowledges the treatment of ordinal Likert ratings as an approximately continuous outcome, clarifies the distinction between the trial-level presentation and the session-level evaluation structure, and provides a DOI-backed archival record for reproducibility. The language and presentation have also been improved.

I consider the manuscript suitable for publication in PLOS ONE.

One very minor editorial point may be checked during production: the Results report both an F statistic and a chi-square statistic for the overall model effect—“F(4,8580) = 20.93” and “χ²(4) = 83.70”—whereas the Methods describe only an F-test. The authors should either briefly identify the source of the chi-square test or omit the redundant statistic. This issue does not require another substantive round of revision.

I recommend acceptance.

**********

7. PLOS authors have the option to publish the peer review history of their article (what does this mean?). If published, this will include your full peer review and any attached files.

If you choose “no”, your identity will remain anonymous but your review may still be made public.

**Do you want your identity to be public for this peer review?**  For information about this choice, including consent withdrawal, please see our Privacy Policy.

Reviewer #2: No

**********

# Editor report: Acceptance letter

**DOI:** 10.1371/journal.pone.0338524.r006

**Authors:** Mohammad Salah Hassan

PONE-D-25-61951R2

PLOS One

Dear Dr. Aramaki,

I'm pleased to inform you that your manuscript has been deemed suitable for publication in PLOS One. Congratulations! Your manuscript is now being handed over to our production team.

At this stage, our production department will prepare your paper for publication. This includes ensuring the following:

* All references, tables, and figures are properly cited

* All relevant supporting information is included in the manuscript submission,

* There are no issues that prevent the paper from being properly typeset

You will receive further instructions from the production team, including instructions on how to review your proof when it is ready. Please keep in mind that we are working through a large volume of accepted articles, so please give us a few days to review your paper and let you know the next and final steps.

Lastly, if your institution or institutions have a press office, please let them know about your upcoming paper now to help maximize its impact. If they'll be preparing press materials, please inform our press team within the next 48 hours. Your manuscript will remain under strict press embargo until 2 pm Eastern Time on the date of publication. For more information, please contact onepress@plos.org.

You will receive an invoice from PLOS for your publication fee after your manuscript has reached the completed accept phase. If you receive an email requesting payment before acceptance or for any other service, this may be a phishing scheme. Learn how to identify phishing emails and protect your accounts at https://explore.plos.org/phishing.

If we can help with anything else, please email us at customercare@plos.org.

Thank you for submitting your work to PLOS ONE and supporting open access.

Kind regards,

PLOS ONE Editorial Office Staff

on behalf of

Dr. Mohammad Salah Hassan

Academic Editor

PLOS One
